# The use of mosquito nets in fisheries: A global perspective

**DOI:** 10.1371/journal.pone.0191519

**Published:** 2018-01-31

**Authors:** Rebecca Short, Rajina Gurung, Marcus Rowcliffe, Nicholas Hill, E. J. Milner-Gulland

**Affiliations:** 1 Department of Life Sciences, Imperial College London, Silwood Park Campus, Ascot, United Kingdom; 2 Institute of Zoology, Zoological Society of London, Regent’s Park, London, United Kingdom; 3 Conservation Programmes, Zoological Society of London, Regent's Park, London, United Kingdom; 4 Centre for Ecology and Conservation, University of Exeter, Penryn, Cornwall, United Kingdom; 5 Department of Zoology, University of Oxford, Oxford, United Kingdom; University of Minnesota, UNITED STATES

## Abstract

Free or subsidised mosquito net (MN) distribution has been an increasingly important tool in efforts to combat malaria in recent decades throughout the developing world, making great strides towards eradicating this hugely detrimental disease. However, there has been increasing concern in the natural resource management and healthcare communities over alternative use of MNs, particularly in artisanal fisheries where it has been suggested they pose a threat to sustainability of fish stocks. So far, little evidence has been presented as to the global prevalence and characteristics of MN fishing, limiting global management initiatives and incentives for action across disciplines. We conducted a rapid global assessment of mosquito net fishing (MNF) observations from expert witnesses living and/or working in malarial zones using an internet survey. MNF was found to be a broadly pan-tropical activity, particularly prevalent in sub-Saharan Africa. MNF is conducted using a variety of deployment methods and scales including seine nets, scoop/dip nets, set nets and traps. MNF was witnessed in a broad range of marine and freshwater habitats and was seen to exploit a wide range of taxa, with capture of juvenile fish reported in more than half of responses. Perceived drivers of MNF were closely related to poverty, revealing potentially complex and arguably detrimental livelihood and food security implications which we discuss in light of current literature and management paradigms. The key policies likely to influence future impacts of MNF are in health, regarding net distribution, and natural resource management regarding restrictions on use. We outline critical directions for research and highlight the need for a collaborative, interdisciplinary approach to development of both localised and broad-scale policy.

## Introduction

The distribution and use of mosquito bed-nets (MNs) in at-risk regions is the front line in the fight against malaria, a disease estimated to threaten 3.4 billion people worldwide [1]. A key objective in the Global Malaria Action Plan is to ‘achieve and sustain universal access to and utilisation of prevention measures’ [1]. The majority of the 97 countries currently experiencing ongoing malaria transmission distribute free or subsidised insecticide-treated MNs. Although the larger-scale campaigns are focused on Africa the effort is global [2]. MN distribution campaigns are estimated to have led to 49% of the at-risk population sleeping under insecticide treated nets in 2013, compared to 2% in 2004 [2]. Malarial incidence is estimated to have fallen by 37% globally between 2000 and 2015, with 16 once-malarial countries achieving or maintaining zero indigenous cases. MN distribution has been a major contributor to this success [3]. However, despite this, concerns have been raised regarding unforeseen impacts of the distribution of billions of insecticide treated MNs. MN ‘misuse’ has been of growing concern from an operational viewpoint for the health community, for example with nets used as crop coverings or protection for granaries [4]. One such concern for both natural resource management and health is the use of MNs within artisanal fisheries [5,6]. With at least 154 million MNs estimated to have been distributed in 2015, and similar numbers in previous years [3], it can be surmised that the incidence of MN fishing is potentially very high, and unlikely to decrease without intervention.

Fine mesh sizes (usually ≤3mm) are critical for exclusion of mosquitos, but render MNs used in fisheries almost entirely unselective in terms of small fish. Reportedly high juvenile fish capture rates [6] are coupled with reports of MN use in mangroves and seagrass beds—important nursery grounds for fish [7]. Additionally, the broad availability and low cost of the nets may be leading to increased fishing pressure from additional fishers entering the fishery [8]. Consequently, due to a perceived undermining of conventional fisheries management the practice is widely illegal [6]. Conversely, it is increasingly acknowledged that small fish may make important contributions to food security in artisanal fisheries [9]. Balanced harvest theory suggests that, where exploitation occurs in a balanced fashion across species and life history stages according to their relative productivity, fisheries can be more sustainably managed. This theory would support some use of small-mesh gears [10].

Concerns have been raised that using nets for fishing reduces bed coverage, impacting the effectiveness of anti-malarial campaigns [5,11], though little evidence exists. Additionally, the majority of these MNs are treated with insecticides, commonly Permethrin which is water soluble [5]. The effects on fish populations and ecosystems are unknown. However, increasingly substantiated concerns over the mass distribution of nets causing resistance of Anopheles mosquitoes, a malarial vector, to these insecticides have been raised [12].

Social issues potentially relating to MN fishing (MNF) include localised conflicts over resources [13], high dependence of vulnerable user groups [8] and low institutional capacity for management [14], which in many cases has led to national bans on MNF [8]. These bans may have detrimental impacts on local livelihoods and food security in the short-term, with the most vulnerable bearing the opportunity costs of management. This critical trade-off serves as good motivation for understanding this issue and its specific impacts better for evidence-based interventions. Key questions emerging include: who are the user groups (at a localised scale) and what is their socio-economic status? What are the drivers and impacts of MN use for these groups? At what scale does this fishing occur and how might external actors and market influences affect MNF? Is it socially just to focus management efforts on a gear for which there is no empirical evidence of harm to fish stocks?

There is still limited peer reviewed literature pertaining to global patterns of MNF and what the influence of these freely distributed nets is on the more general use of small-mesh gears, particularly outside Africa. Indeed, we could find only brief mentions of MN use in fisheries of India, Bangladesh, Timor Leste and the Solomon Islands [15–18]. Within this literature the reported user demographics, methods, perceived impacts and extent of MN fishing (if mentioned, which was rarely) are variable. Small-scale case studies are beginning to emerge with localised policy implications, which have also served to highlight the potential cultural and geographical heterogeneity of the issue in terms of both ecological and health impacts: McLean et al., 2014 [5] along the Democratic Republic of Congo’s side of Lake Tanganyika; and Bush et al., 2016 [8] in coastal Kenya. In addition, both studies have alluded to a possible underappreciation of the prevalence and scope of MN use in fisheries, particularly in sub-Saharan Africa [5] but also anywhere where MNs are distributed globally. Recent high profile and widely shared media articles (e.g. [6]) suggest that there may now be a platform from which this issue can begin to be discussed. However, the lack of a global perspective on the extent and characteristics of MN fishing may preclude the addressing of the higher-level, trans-boundary and multi-stakeholder policy implications of MN fishing (for example for malaria control strategies by global health organisations).

To date, assumptions about the fisheries impacts of MNF in the peer-reviewed literature have largely been based on current scientific paradigms around natural resource exploitation and socio-ecological dynamics, namely the need for size-selective and effort-based management. However, a lack of real-world empirical evidence on the size and species caught, coupled with recent questions about the universal validity of size-based management posited through balanced exploitation theory [19], puts these assumptions in question. Critically, whilst on aggregate the literature suggests that MNF is widely distributed, with numerous mentions of the activity within studies focused on other topics, there has been no empirical investigation as to the actual extent and prevalence of MNF (S1 Table). There is an urgent need for better information on the global patterns of MNF.

In this article, we address this need, by providing a rapid assessment of the current state of awareness and perceptions about MN fishing at a global scale as an initial scoping exercise to generate some indication of the prevalence and nature of MNF. We use an online survey of predominantly charity-sector workers to undertake a preliminary and broad-scale investigation in to the variability in who, how and why people use MNs for fishing, setting the scene for future detailed investigations at a finer resolution. We also highlight the implications of our findings for policy and MNF management.

## Methods

### Online survey

An online survey was made available in English and French between 4/6/15 and 14/8/15 using the Qualtrics Survey Software [20]. Information regarding MNF was requested from anyone living or working within any area of malarial risk, either coastally or close to bodies of water used for fishing at any scale, with a focus on obtaining responses from relevant stakeholders in the fisheries management, public health, conservation and development sectors. By sampling these groups we deemed that relevant and detailed responses would be more likely, detail would be more reliable based on respondent experience and the survey would benefit from snowball distribution to relevant networks. Additionally, we deemed the online survey method to be the fastest and most cost-effective way of obtaining responses. Although this method excludes those without internet access and can suffer limitations in scope and uptake, this sampling strategy and target audience was used to attempt to maximise access (both in terms of internet connection and language), generate good quality data and rapidly glean a global perspective on an issue which is rarely a primary focus of any of these sectors.

Qualifying questions on, for example, time the respondent has spent at the location, organisational affiliation and associated role were used to gauge levels of confidence in observations. Two survey options were available: for individuals whose experience was predominantly fisheries/conservation/ecology focused, and for those whose experience was predominantly development/health focused. The latter omitted questions for which a higher level of ecological knowledge was necessary. We requested observations of MNF at the ‘*village level*’ or equivalent but also accepted were ‘*areas of coastline*, *river*, *lake*, *fishing location or region’* if later geographically defined. Respondents could provide more than one observation by completing the survey for each location where they had first-hand, personal knowledge of MNF. We solicited both negative and positive observations of MNF in order to reduce positive bias. We included duplicate observations at given locations if additional information significant to the study objectives was provided.

We promoted this survey to relevant respondents through the authors' own networks, relevant mailing lists, newsletters, conference delegate lists and direct targeting of relevant individuals and subsequent networks through internet searches. Social media outlets Facebook and Twitter were utilised extensively with all authors’ affiliated organisations participating and expanding the reach. Every effort was made to ensure geographical representation and to limit potential bias from factors such as prevalence of NGO activity in an area. Whilst negative observations are not conclusive evidence of absence, some confidence is afforded by the general visibility of MNF as an activity. Where deemed necessary and feasible, we contacted respondents directly for additional detail, reports, papers and photographic evidence.

### Ethics

Ethical approval for this research was granted through the Imperial College’s MSc in Conservation Science’s research ethics process, involving formal review and approval by a committee of Faculty members. All responses were anonymous unless the respondent chose to identify themselves. No questions required information which could identify individuals engaging in MNF and all personal information relating to respondents was available only to the authors. Quotes are only used with consent from respondents. Detailed location data (at a resolution finer than 1 degree or with descriptive information) are available only on application to the authors and based on the undertaking that fine-scale locations are not identified in subsequent analyses.

## Results

Ninety four observations of presence and 36 observations of absence of MNF were received from 113 respondents. Here we explore only presence observations, in order to guard against bias, but the absence records are given for information in the Supplementary Material. Fifty seven observations were given from those working in the conservation and ecology sector, 17 from development and health, 17 with a fisheries focus and 3 in relevant commercial or tourism roles (Table 1). One hundred and twenty six observations included specific location information.

**Table 1:** Presence observations of MNF by region and work sector of respondent.

Work sector	Americas	Asia	East Africa	Oceania	West & Central Africa	Grand total
**Conservation**	2	7	36	2	10	**57**
**Development & Health**		1	13		3	**17**
**Fisheries mgmt.**		3	11	1	2	**17**
**Commercial & tourism**			3			**3**
**Total**	**2**	**11**	**63**	**3**	**15**	**94**

### Spatial and temporal prevalence of MNF

**Fig 1:**
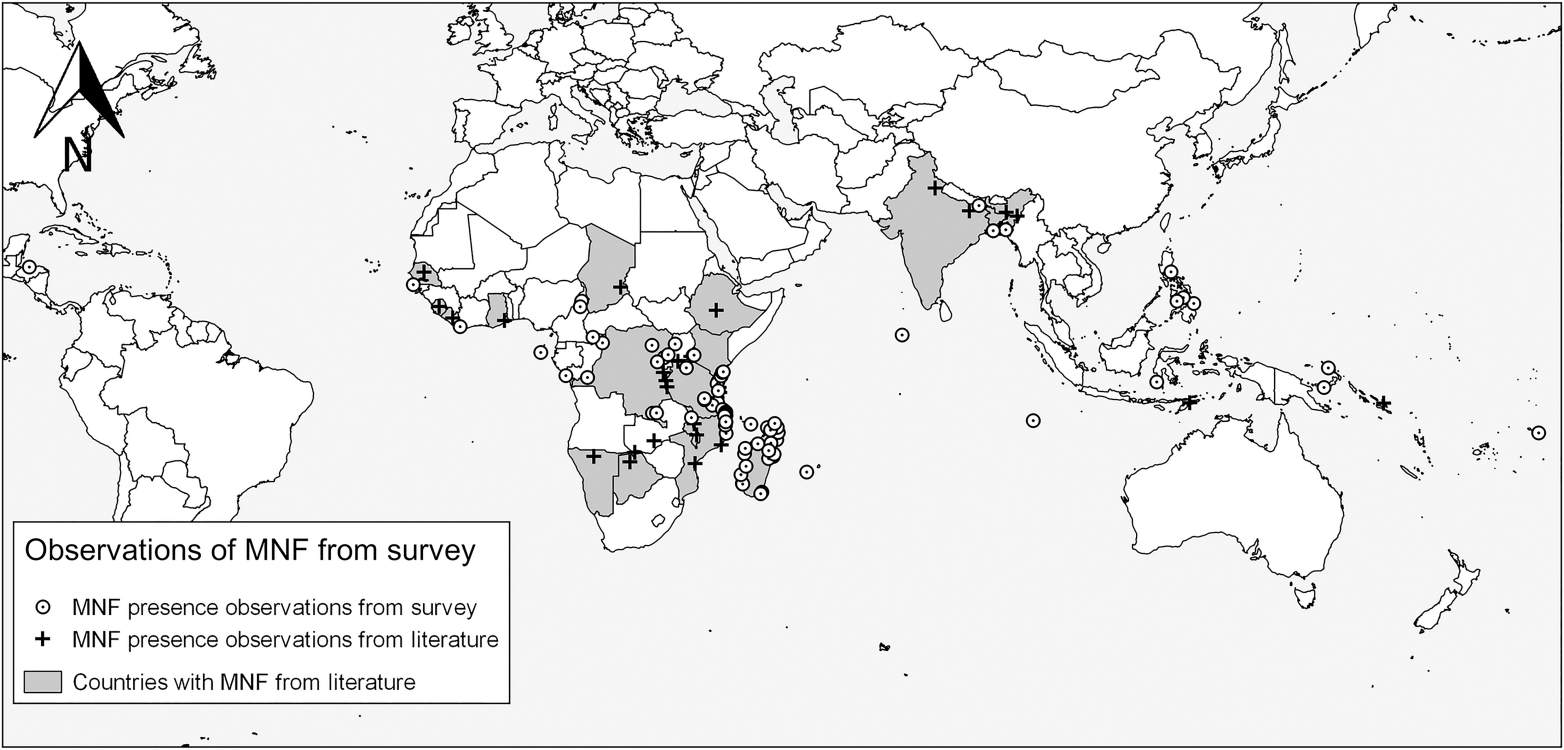
Global map of survey responses showing presence reports of MNF from the survey and confirmed locations from the existing literature.

Reports of MNF presence came from 26 countries across all equatorial continents, 16 of which (74 responses) were in sub-Saharan Africa (Table 1). Results highlighted the presence of MNF in 18 countries for which there were no previous records of MNF in the peer-reviewed literature (Fig 1). Eight of the countries with records of MNF in the literature were not represented in our survey. Globally, 66% of location observations were in marine environments and 34% in freshwater.

Reports from Asia were clustered in the Philippines and Bangladesh (S1 Fig) and were predominantly coastal, with the exception of Nepal. Papua New Guinea and American Samoa had the only observations in the Oceania region.

Observations of the presence of MNF from the Africa region were heavily skewed towards the sub-Saharan, Indian Ocean nations with an additional cluster of observations around the African Great Lakes (Fig 1). In Madagascar, 16 observations (the highest of any country) covered much of the coastline, as well as Lac Alaotra, the largest freshwater body. Observations were also made inland large distances from substantial bodies of water in riverine environments. Only two presence observations were made in the Americas–in Honduras and Ecuador.

**Fig 2:**
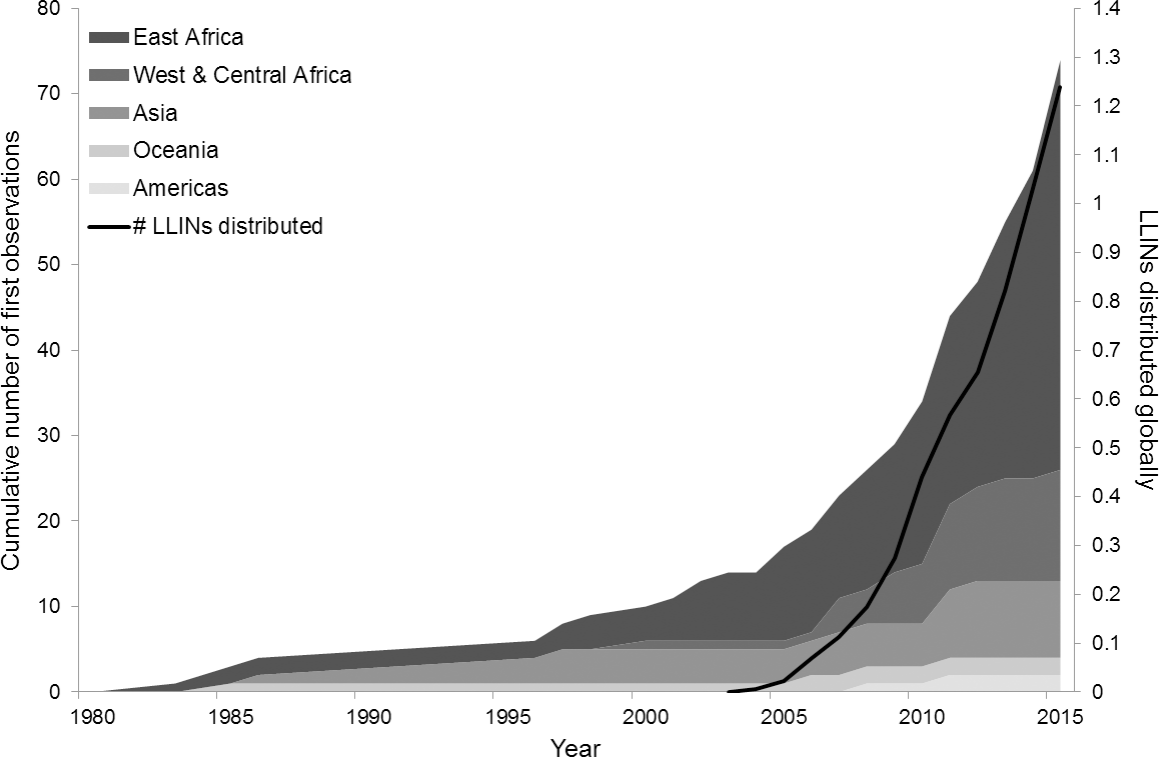
Cumulative first observations of MNF by region. Black line represents Global cumulative number of Long Lasting Insecticide-treated nets (LLINs) distributed since launch of Roll Back Malaria Programme, net data sourced from The Alliance for Malaria Prevention Net Mapping Project (2004-present) [21].

Seventy four observations included the first year in which they observed MNF at that location. A cumulative frequency curve (Fig 2) shows a steady rise in first observations beginning in the mid-1970s and continuing until the present day, corresponding closely with the Alliance for Malaria Prevention’s net distribution figures which are available from 2004 [21]. This trend holds across Asia, East Africa and West and Central Africa.

### Biome reporting rates and habitat use

One hundred and eight reports of biomes associated with MNF were given: 59% coastal, 13% lacustrine, 25% riverine and 3% wetlands. Of 177 responses for specific habitat use the majority (31%) report MNF use on beaches/sandflats, twice as many as in seagrass beds and mangroves (Table 2). MNF was reported across all marine and freshwater habitats in both Africa and Asia (S4 Fig).

**Table 2:** Proportion of responses reporting MNF activity in different habitats globally.

Habitat	Proportion total obs (n = 177)
Beach/sandflat	0.31
Seagrass bed	0.15
Mangrove	0.14
River	0.13
Pelagic	0.08
Lake	0.07
Local stream	0.07
Coral reef	0.05

### Gear characterisation, deployment and users

The majority of respondents reported that deployment took place on foot (60%, n = 115), but with canoe use also featuring prominently (29%), particularly in W&C Africa. MNF from sail and motorised boats was also reported across all three regions (S7 Fig).

Four predominant MNF methods were identified from the literature, and clarified further by our survey: Single-net use, with nets largely unaltered and operated by individuals or pairs, dominated across all regions (53%, n = 105), followed by multiple nets sewn together for use by small groups of fishers (34%), then use as a cod end of larger seine nets (10%) and finally just three reports of insecticide fishing, the details of which remain unconfirmed but where in one case additional DDT is thrown in to the water along with the MNs. Numerous other methods were described by survey respondents, including a number of trap designs, ‘scoop’ or ‘dip’ nets, and the use of static ‘set’ nets used to funnel fish, sometimes with photographic evidence (S7 Fig).

The reported frequency of engagement in MNF varied significantly across demographic groups, with women most commonly reported as engaging in MNF ‘often’, and men, children and the elderly most commonly reported as engaging in MNF ‘sometimes’ (S5 Fig, X-squared = 38.94, df = 6, Cramer’s V = 0.27, p = <0.001). Thirty-five percent of observations reported those engaging in MNF locally to be experienced fishers, 43% part time and 21% inexperienced fishers.

### Species caught

Response rates were low for questions relating to taxonomic and maturity composition of MN catch; anything speculative was removed from the dataset and only confident instances retained. Thirty-eight families of fish were identified as present in MN catch across methods, habitats and regions; 7 freshwater and 33 marine (S2 Table). Additionally, general reports of squid, crabs and shrimp were made, with the last of these identified as a significant component of coastal MNF catch. Particularly high value species targeted included seahorses in Papua New Guinea (Chinese Traditional Medicine market-driven).

Fifty-nine respondents cited presence of juveniles in MN catch. Of 69 respondents identifying important targeted taxa at a generalised level, 29 reported targeting of reef-associated fish, 13 pelagic/neritic species, 6 molluscs and 14 crustacea (S2 Table). Species that were frequently reported as significant targets for MN fishers were:

- Marine shrimp species: all regions.
- The Common silver biddy (*Gerres oyena* and similar species): known as ‘Sala’ in E. Africa.
- Milkfish (*Chanos chanos*): both African and Asian fisheries, often targeted for wild-caught fry aquaculture.
- Silver cyprinid (*Rastrineobola argentea*): commonly known as ‘Dagaa’ or ‘Omena’ in the fisheries of Lake Victoria.
- Lake Malawi sardine (*Engraulicypris sardella*): known as ‘Usipa’, fisheries of Lake Malawi.

### Perceptions of MNF drivers and impacts

The majority of respondents observed MNF catch to be locally important for both consumption and sale (66% n = 87). Additionally, the use of MNF catch as bait for other gears was identified, along with large scale sale to animal feed companies in Madagascar.

**Fig 3:**
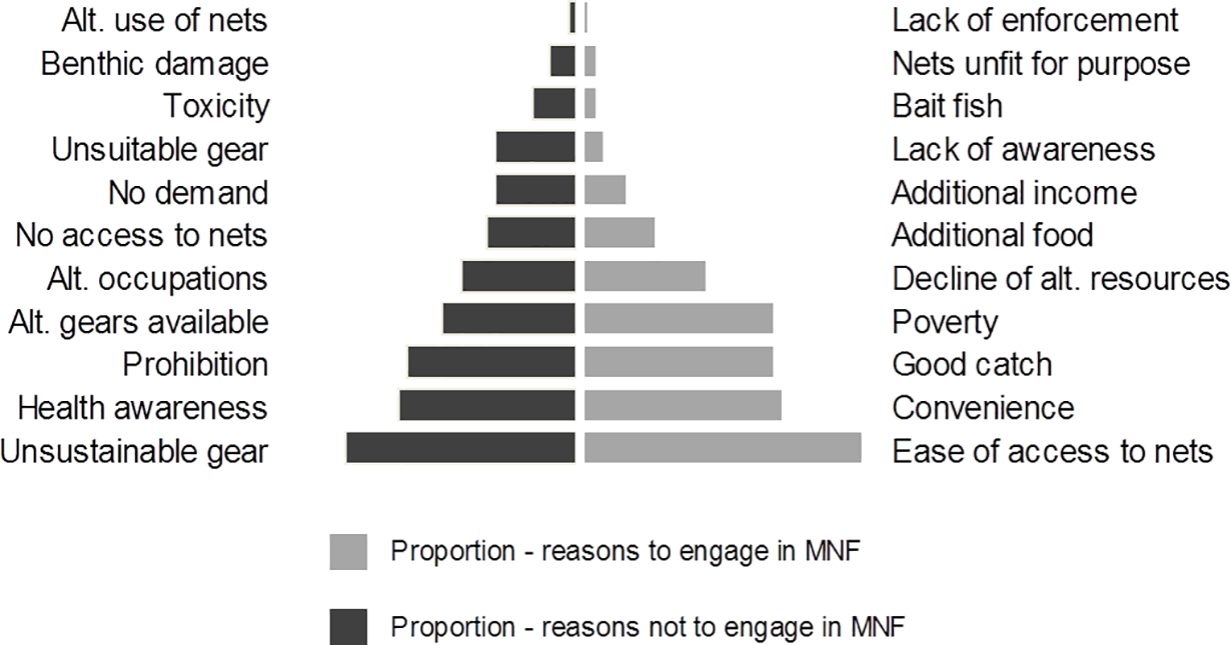
Proportion of observations citing various drivers for people engaging and factors that may influence people not to MN fish.

Perceived drivers of MNF were dominated by the incentives (pull factors) of readily available nets, convenience of the method/catch and good catch, along with the forcing, push factor of poverty, followed by perceived declines in alternative resources (Fig 3). Qualitative responses of note include: one report of MNs deliberately distributed to communities in Papua New Guinea by Chinese traders targeting seahorses for the Chinese Traditional Medicine market, and another report of MNF in Madagascar driven by demand from animal feed companies targeting forage fish.

Respondents speculated that people may choose not to fish with MNs due to perceptions of unsustainability, risk of mosquito-borne diseases, prohibition, and preference for alternative occupations. A lack of access to MNs ranked sixth in this list, suggesting this is not often a limiting factor and nets are considered widely available.

## Discussion

Concerns over the use of MNs in artisanal fisheries have been expressed in disparate locations in the peer-reviewed literature since the early 2000s but have thus far lacked formal investigation at a global scale. It has been proposed that the impacts of MNF are likely to relate to both the selectivity of the fine mesh nets, and also to the potential for increased fishing pressure resulting from the nets' availability and ease of use. Therefore it is important to begin to understand both the characteristics of the fishery and also the current distribution and prevalence of MNF. This study aims to gather information on experts’ awareness and perceptions of MNF in order to broadly characterise MNF globally, and gain valuable insights from those witnessing the activity in order to highlight research needs and catalyse debate across stakeholder groups.

### Characterising the global prevalence of MNF

A critical question posed by the public health community when engaged on the issue of MNF and whether or not a policy response is required is: how widespread of an issue is it? Is it just isolated pockets on a few lakeshores or engaged in by multiple people in multiple communities? Here we have aimed to answer these questions to a degree necessary to catalyse an appropriate response. MNF is widely represented across all equatorial regions; our survey results confirm presence of the activity in most regions classified as ‘at risk’ areas for malarial transmission, save for European and Middle Eastern regions. This distribution correlates with regions where efforts to supply free and/or subsidised MNs as part of anti-malarial efforts are particularly pervasive [3]. Although it is unwise to infer absolute levels of prevalence of MNF by region from a non-random survey, recent suggestions of rapid increases in MNF activity in the East African region in the media, peer reviewed and grey literature [5,6,8,22] appear to be supported by our results, with a high concentration of reports both coastally and around the African Great Lakes. However, high response rates from Mozambique and Madagascar may be influenced by well-established networks of NGOs operating in the region that were able to distribute the survey widely.

The cumulative frequency of first observations (Fig 2) appears to align with the cumulative number of nets distributed globally; according to the Alliance for Malaria Prevention (LLINs only are presented here, other nets have been distributed but not on a similar scale). The launch of WHO’s Roll Back Malaria (RBM) programme in 1998 had the goal of unifying public and private efforts towards tackling malaria globally. Goals were set for universal coverage of bed nets for those living in at-risk areas. A few years later large scale distribution programmes began globally, but with a particular focus on sub-Saharan Africa [3]. These results may therefore reflect the rise in MN distribution campaigns under the WHO malaria programme. However, they should be treated with caution as just ten respondents had witnessed the introduction of the activity personally (as opposed to on their arrival in an area). There are also well documented issues of recall accuracy and the fact that observer presence has increased in recent years stems from increased NGO activity in many regions, whether health or environment-focused.

Differences in reported prevalence of MNF in Africa and Asia point towards a possible link between MNF and MN-distribution efforts. Net distribution efforts in Asia are considerably lower than in sub-Saharan Africa. Although internal national efforts exist, 82% of international investment has been directed to Africa [3], so net availability may be a contributing factor. The activity may also have a different level of visibility in Asia where small mesh nets in general are more common and MNs may be indistinguishable from other materials. The limited information gleaned for the Americas and Oceania, despite confirming presence of MNF from at least two sites in each region, does not support any broad inferences as to prevalence.

Although the limited peer reviewed literature incorporating information on MNF is largely focussed on freshwater environments [5,11,23,24], our survey suggests that MNF is widespread and frequent in marine environments. We can also infer that where MNF has been reported to occur (marine or freshwater) it is a frequent and perennial activity. This could indicate that MNF has become part of daily livelihood and/or consumption portfolios for at least certain user groups. Consideration of livelihoods is therefore of great importance when designing interventions/policy options.

### Variability in MNF characteristics

Most deployment appears to remain small-scale, with the use of one or a few nets sewn together as small-scale seine nets in shallow-water environments. Use on coral reefs was reported infrequently and anecdotal information suggests that MNs are largely unsuitable for this environment due to frequent tearing.

Shallow water environments such as seagrass beds, sand flats and mangroves can host large biomasses of fish [25] which may have been less accessible and/or desirable before the advent of MNF. Although similar, traditional fishing methods using cloth are documented and associated with MNF communities in the survey, such as ‘Tandilo’ in East Africa, [8] their efficacy is likely to be lower than MNF but precisely what impact the addition of MNs may be having cannot be inferred from this study. As long as people have a use or market for the associated species then it can be inferred that MNs may confer an important advantage to users either as an additional gear or an alternative livelihood choice. Use in shallow water may also mean that MN fishers can continue to engage in traditionally widespread gleaning activities for resources such as octopus and molluscs concurrently.

### Implications for management

Our results support the suggestion that MNF is a highly accessible fishing activity which doesn’t require the skill, knowledge, vessels, opportunity costs or capital investment necessary for other fishing gears. Looking at motivations from a ‘push’ or ‘pull’ perspective, the perceived drivers cited by observers are dominated by pull factors; positive reasons why one would choose to MN fish, and underlying this was the predominant push factor of poverty. The real variation in drivers, important to intervention design, may lie in the user groups. For those users already classified as ‘vulnerable’ or ‘marginalised’, MNF offers an opportunity to reduce this vulnerability. In many traditional artisanal fisheries women play a vital supporting role in processing and sales of fish, and are also involved in gleaning [26]. However, in many places women are still considered a marginalised and vulnerable group because of cultural norms that limit their access to fisheries [27]. MNF represents a more efficient method for traditional gleaning that could generate higher returns. Our data also suggest that the experience levels of fishers are variable and that occupational multiplicity is common amongst those engaging in MNF. This may be particularly important for traditional farmers and natural resource users driven off their land by climate change or changing land uses and resettlements [28]. For example, the Giriama in coastal Kenya were settled in Mida Creek as a result of resettlement and took up MNF [8].

If MNF becomes increasingly attractive economically, either due to other fisheries declining or development of new markets, this could increase male engagement in cases where MNF is currently deemed ‘women’s work’ (e.g. seaweed farming in Tanzania, at first undertaken predominantly by women, was later dominated by men once its commercial value was deemed sufficient [29]). Market-based factors such as the ever-growing reach and size of the Chinese Traditional Medicine and animal/aquaculture feed markets may drive these changes.

Respondents’ perceptions of reasons not to engage in MNF most commonly alluded to fishers' perceptions of the unsustainability of the practice. These perceptions are of course from people external to the fishery; in reality this motivation is likely to vary widely between user groups depending on an interaction between people’s perceptions, ecological understanding and the degree with which they engage/rely on the wider fishery. Awareness of the health benefits from correct use of MNs is second-most cited and there are examples in the literature of increased awareness-raising impacting levels of alternative MN use [5,30]. These efforts are likely to be impacted by overall availability of nets. For example Bush et al., (2016) found that in Mida Creek, Kenya there was unlikely to be a trade-off between malaria prevention and MNF as nets were so readily available.

This study was not an empirical investigation into the sustainability of MNF. We therefore do not present conclusions on the ecological impacts of MNF, but our findings do give useful preliminary insights to guide future research. The observations of catch composition and juvenile capture do lend us some critical first insights that may support and oppose current concerns, but certainly illustrate the need for further investigation. The diversity of marine MNF catch is likely to be due to the utilisation of habitats such as seagrass beds and mangroves for MNF, which are important nursery grounds for both pelagic and reef-associated species [7]. The range of families and functional/trophic groups reportedly caught in MNs in the marine environment is of concern for selective fishing management regimes. Some species and/or life history stages targeted are those for which there was limited former demand (e.g. juvenile *Gerres oyena*), potentially expanding fishing impacts, but also providing a potentially valuable new resource. Fish also often occupy multiple niches at different life stages. Exploitation of a species at an increasing number of these life stages could be disruptive at the ecosystem level, or may conversely contribute to a more balanced harvest-type scenario with increased overall yields if managed appropriately.

Reports of high juvenile capture rates are also of concern in both marine and freshwater environments where conventional management is a goal. Although it is impossible to verify the specific biological knowledge of every respondent, enough respondents were able to identify fish to family or species level, and verify juvenile capture at this scale to warrant investigation. Within the literature this is the biggest concern pertaining to MNF due to the undermining of size-selective management [5], and the potential for growth and/or recruitment overfishing of stocks that are relied upon by other user groups. However, size-selective management, as well as being generally inappropriate and prohibitively difficult to implement in artisanal scenarios, is no longer the predominant accepted management discourse. Particularly where food security is the biggest concern and where wet weight of protein may be prioritised over rents, balanced harvest is increasingly thought of as a more pragmatic approach to management [31] and to achieving new goals of ecosystem-based management [10]. It is worth considering, therefore, the critical importance of understanding the user groups for MNF and their vulnerability alongside empirical assessments of their impacts on a fishery. Strong arguments exist for an underestimated importance of the harvesting of small bodied fish in subsistence communities [19]. MNF investigations should not disregard a potential synergy with ecosystem-based management goals and benefits posited by balanced harvest theory including increased protein provision and positive contributions to nutrition through micro-nutrients, particularly for children [32]. Small mesh nets may play an important role in optimising yields (albeit of potentially low-economic value catch by western standards) in a balanced harvest scenario which, coupled with the accessibility of MNF, could contribute in a significant way to social equity and overall food security. Though this consideration of social equity is deeply complicated by the health element of the MNF issue, the distribution of nets for anti-malarial purposes hinging importantly on collective compliance, it will nevertheless be critical to development of effective management interventions.

External to the debate over the direct impacts of MNF on the target fishery resource, however, reports of habitat damage are worrying in fragile habitats such as seagrasses where regular seining and trampling may have long term impacts. Also of concern are emerging market-based drivers, such as the Chinese Traditional Medicine and animal feed examples which are new to some of these areas, wherein external influences are introduced and environmentally and economically destructive behaviours encouraged.

### Future directions

The aim of this study was to set the stage globally and identify the current state of, and gaps in, knowledge to guide future research in this novel arena. Although this is a global review, the issue requires localised research. Therefore, we advocate for a portfolio of case studies with which to inform policy at the local level, while providing broader insights, aiming to:

- Identify and map linkages between areas where MNF currently occurs/is expanding and potential driving influences such as prevalence and characteristics of net distribution, estimates of net ‘availability’ and net ‘leakage’, resource management capacity, broad fishery types and gear availability/accessibility. This will require a broad cross-disciplinary approach including data and knowledge sharing.
- Empirically assess ecological impacts across the scale of MN use. Studies need to qualify and quantify the direct impacts faced in terms of overexploitation and interactions of indirect impacts such as habitat damage. Predictive modelling coupled with empirical studies would allow us to understand how this might impact fisheries more broadly.
- Understand specific drivers of MNF for different user groups at a local level, being mindful of varying vulnerability and the potential for indirect drivers of MNF within coupled socio-ecological systems, particularly emerging market forces.
- Determine the level to which MNF has become entrenched as a livelihood and/or subsistence activity within communities and user groups, and therefore the potential difficulties of reducing MNF.
- Investigate how MN distribution efforts interact with MNF e.g. is growth in MNF correlated with specific net characteristics; do free vs. subsidised policies have an impact on MNF levels and if so why; does temporal spacing between re-distributions impact MNF and if so why; what effect might net retrieval schemes have?
- Conduct assessments of localised institutional capacity for management, both formal and informal, with a focus on inclusion of vulnerable groups.
- Collate and assess examples of interventions which have addressed the drivers of MNF, not merely reduced incidence, such as education and awareness programs, livelihood interventions, integrated gear management efforts.

All of these research strands need to be pulled together to inform a fully collaborative interdisciplinary approach to the issue. The perception data presented here indicate that the drivers of MNF are complex and may be influenced by policy change in both fisheries management and healthcare interventions. Therefore we hope that this research can act as a catalyst for collaboration between the health, fisheries management and conservation sectors. MNF is global, expanding and complex. Whilst strides are made to eradicate malaria, mitigation of unwanted and unforeseen consequences to natural resource sustainability must be of priority to avoid additional harm to developing nations’ fishing communities and indeed potentially negative feedbacks on human health. Additionally, it will be important not to lose sight of the ecosystems and biodiversity at stake, making MNF a conservation issue. Biodiversity conservation strives for a ‘do no harm’ approach to interventions, increasingly seeking to marry development and conservation towards mutual sustainability goals. We would advocate for similar principles being adhered to in antimalarial efforts and in an interdisciplinary approach to this problem, seeking collaborations toward outcomes that minimise social and environmental impacts in pursuit of malaria control.

## Supporting information

S1 Fig Map of survey results, Asia. (PDF)

S2 Fig Map of survey results, Africa. (PDF)

S3 Fig Global map of fishing frequency and seasonality. (PDF)

S4 Fig Regional MNF habitat use. (PDF)

S5 Fig Frequency of engagement by demographic group. (PDF)

S6 Fig Deployment and method use by region. (PDF)

S7 Fig Summary of additional respondent information. (PDF)

S1 Table Predominant peer review literature for MNF. (PDF)

S2 Table Identified fish families. (PDF)

S1 Text Survey questionnaire. (PDF)

S2 Text Additional MNF demographics and legislation results. (PDF)

S1 File Raw online survey data. (CSV)
