# Supplementary figures and images for: The use of mosquito nets in fisheries: A global perspective
Source: PLoS One. 2018 Jan 31;13(1):e0191519. doi: 10.1371/journal.pone.0191519 (PMC5791988; doi:10.1371/journal.pone.0191519)

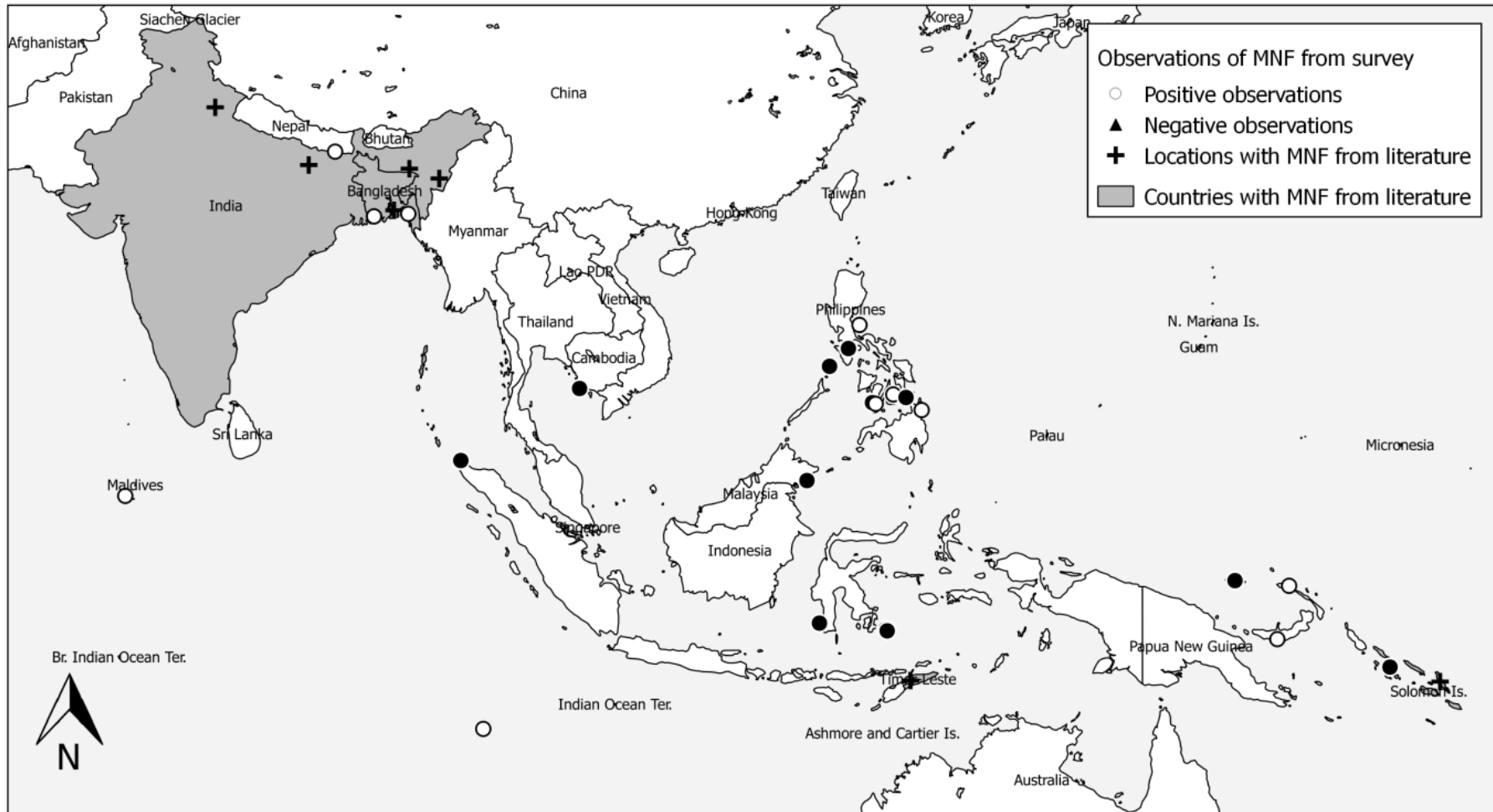

**S1 Fig. Map of survey responses in Asia and Oceania showing positive and negative reports of MNF**

Supplement: S1 Fig — (PDF) [file pone.0191519.s001.pdf]

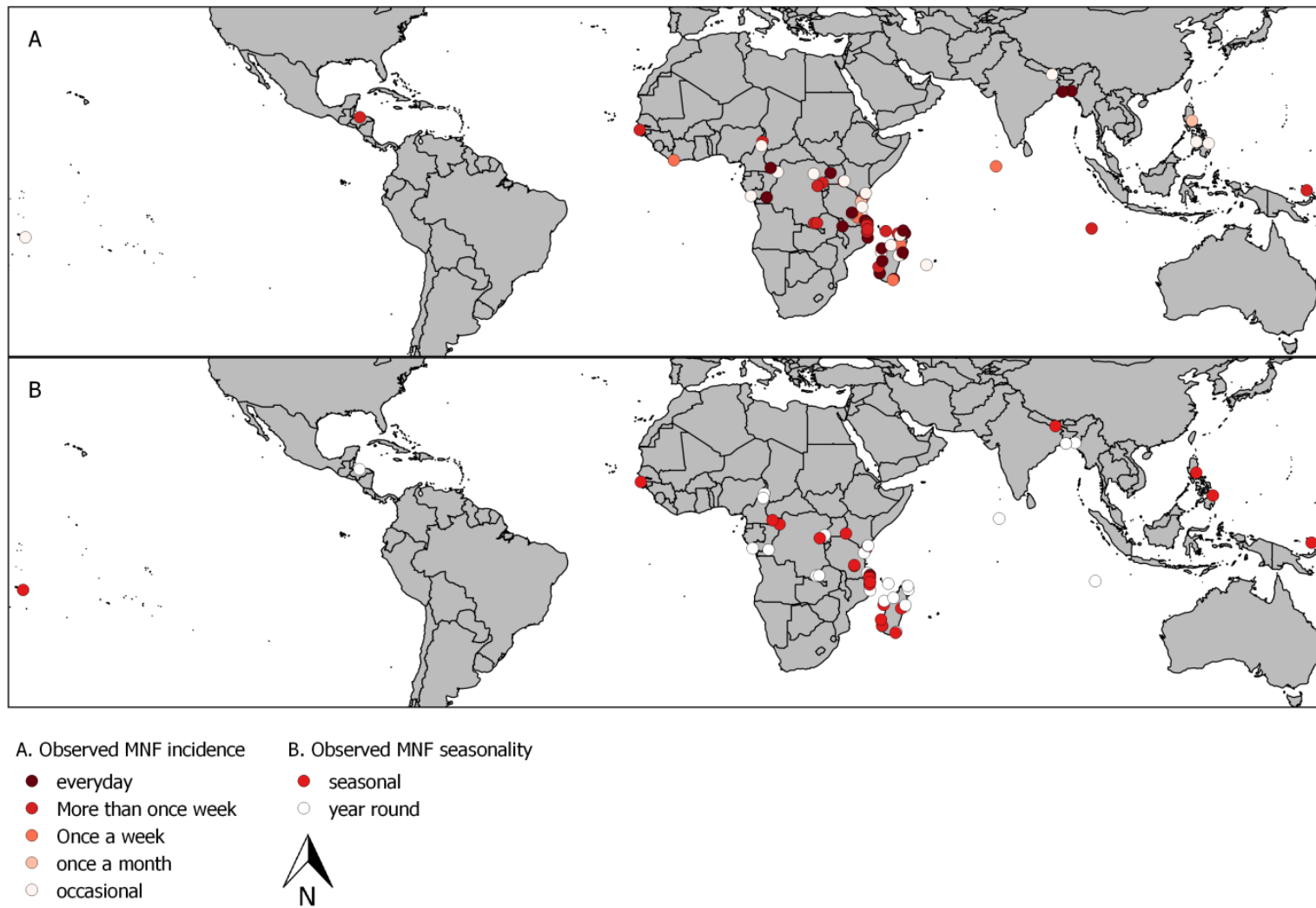

**S3 Fig. Observed global incidence frequency and seasonality of MNF from survey responses**

Supplement: S3 Fig — (PDF) [file pone.0191519.s003.pdf]

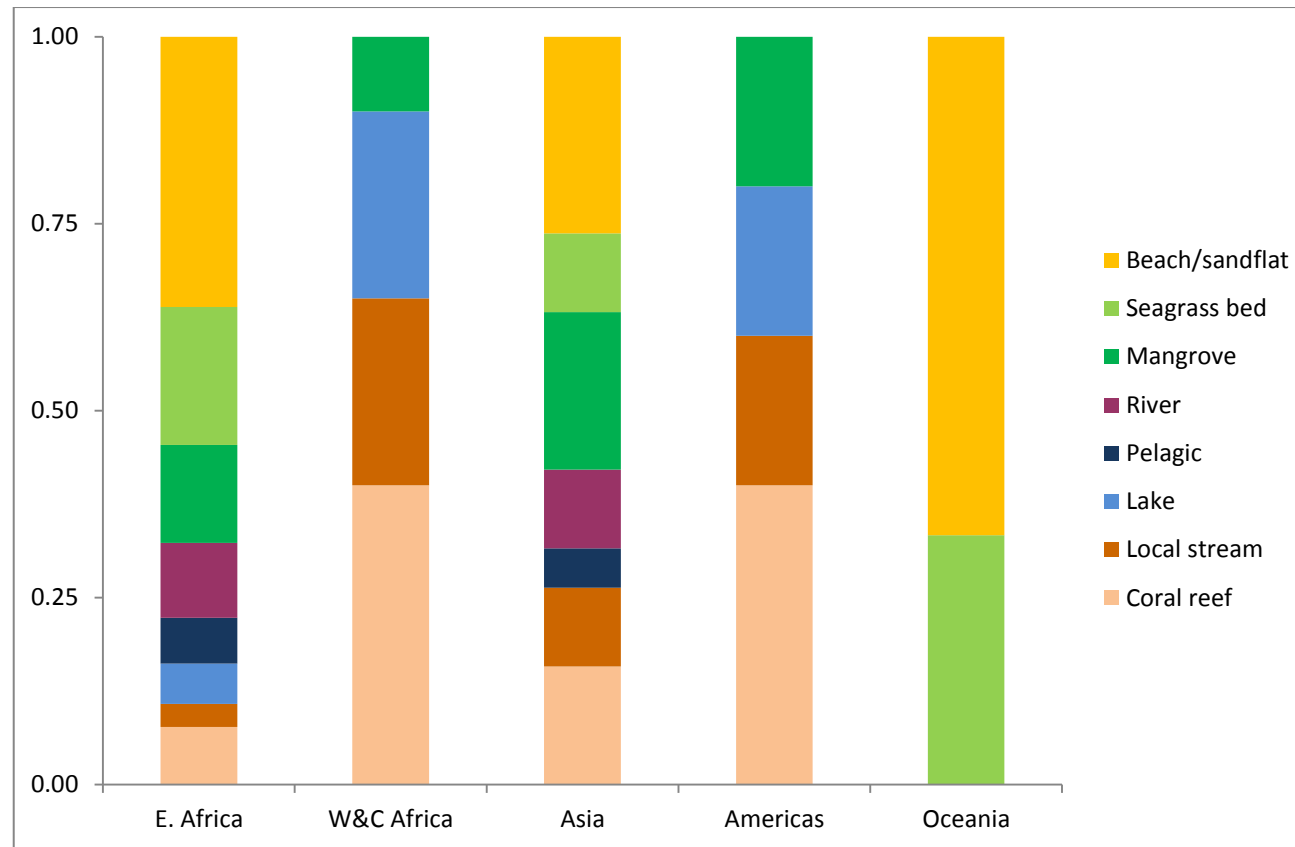

**S4 Fig. Proportion of habitats utilised by MN fishers reported by region.**

Supplement: S4 Fig — (PDF) [file pone.0191519.s004.pdf]

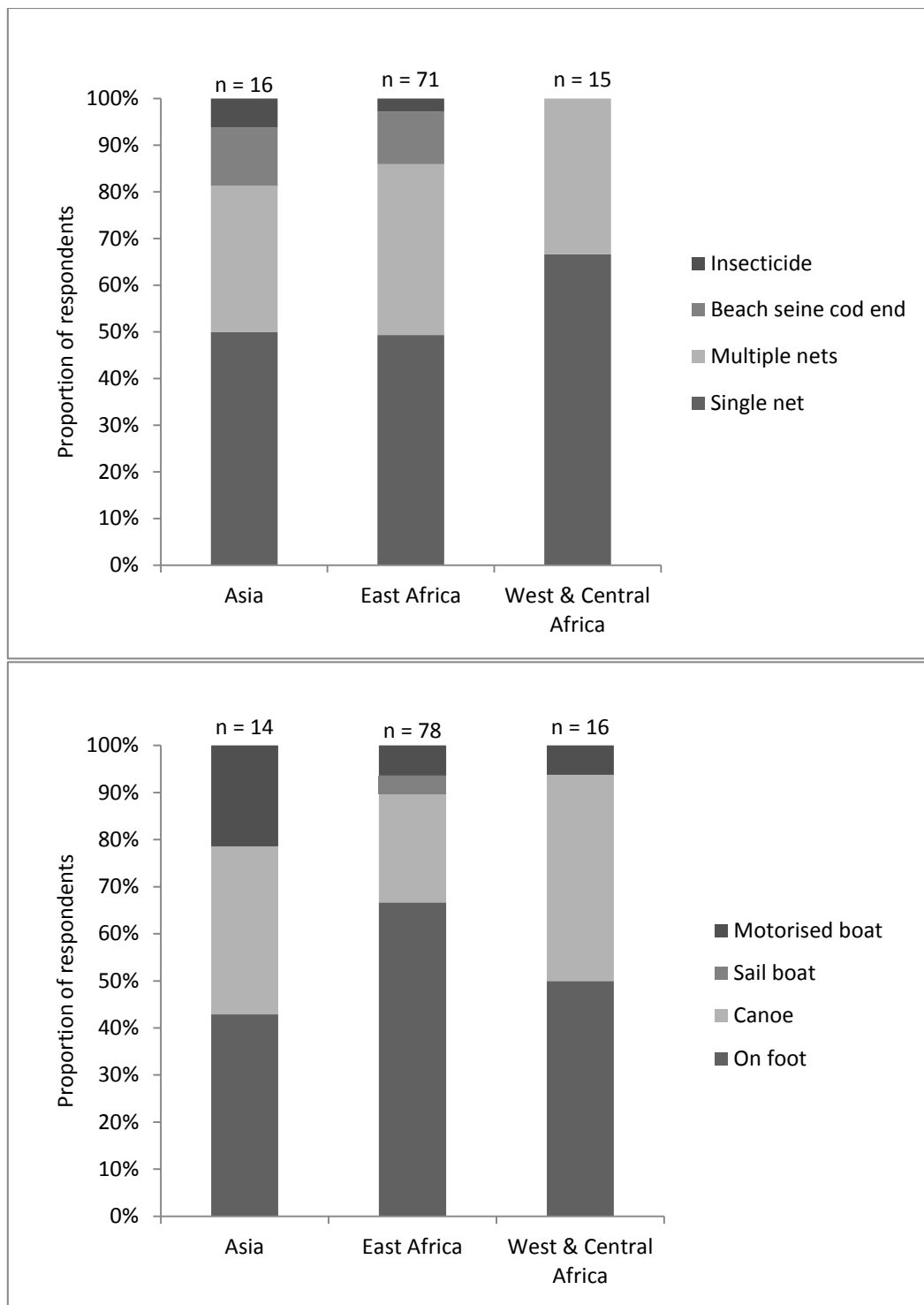

**S6 Fig. Proportion of respondents reporting various modes of deployment for MN fishing.**

Supplement: S6 Fig — (PDF) [file pone.0191519.s006.pdf]
