# Supplementary material for: The use of mosquito nets in fisheries: A global perspective
Source: PLoS One. 2018 Jan 31;13(1):e0191519. doi: 10.1371/journal.pone.0191519 (PMC5791988; doi:10.1371/journal.pone.0191519)
Supplement: S5 Fig — (PDF) [file pone.0191519.s005.pdf]

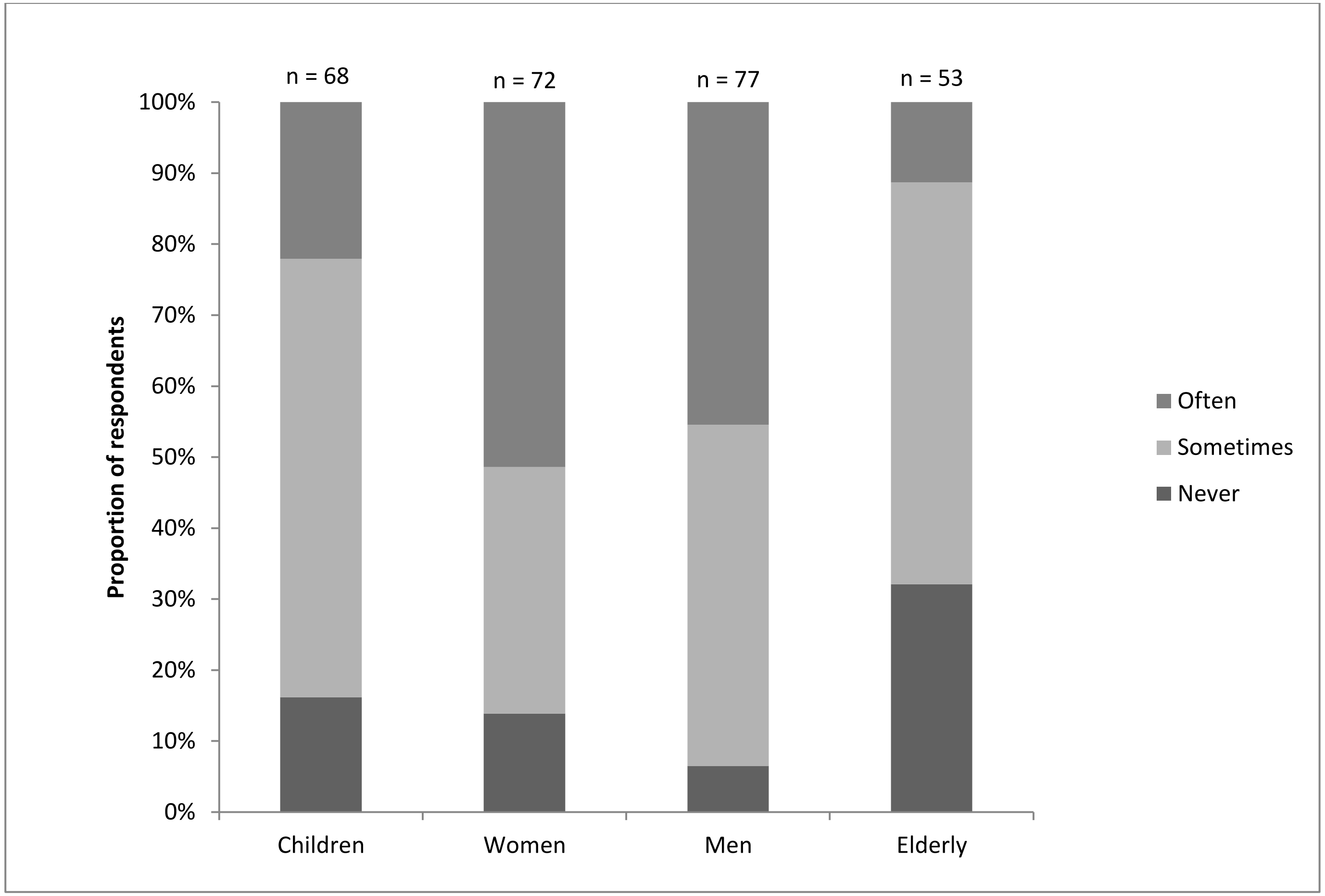

S5 Fig. Demographics of proportion of the observed population engaging in MNF either 'often', 'sometimes' or 'never'.
