# Supplementary material for: The use of mosquito nets in fisheries: A global perspective
Source: PLoS One. 2018 Jan 31;13(1):e0191519. doi: 10.1371/journal.pone.0191519 (PMC5791988; doi:10.1371/journal.pone.0191519)
Supplement: S7 Fig — (PDF) [file pone.0191519.s007.pdf]

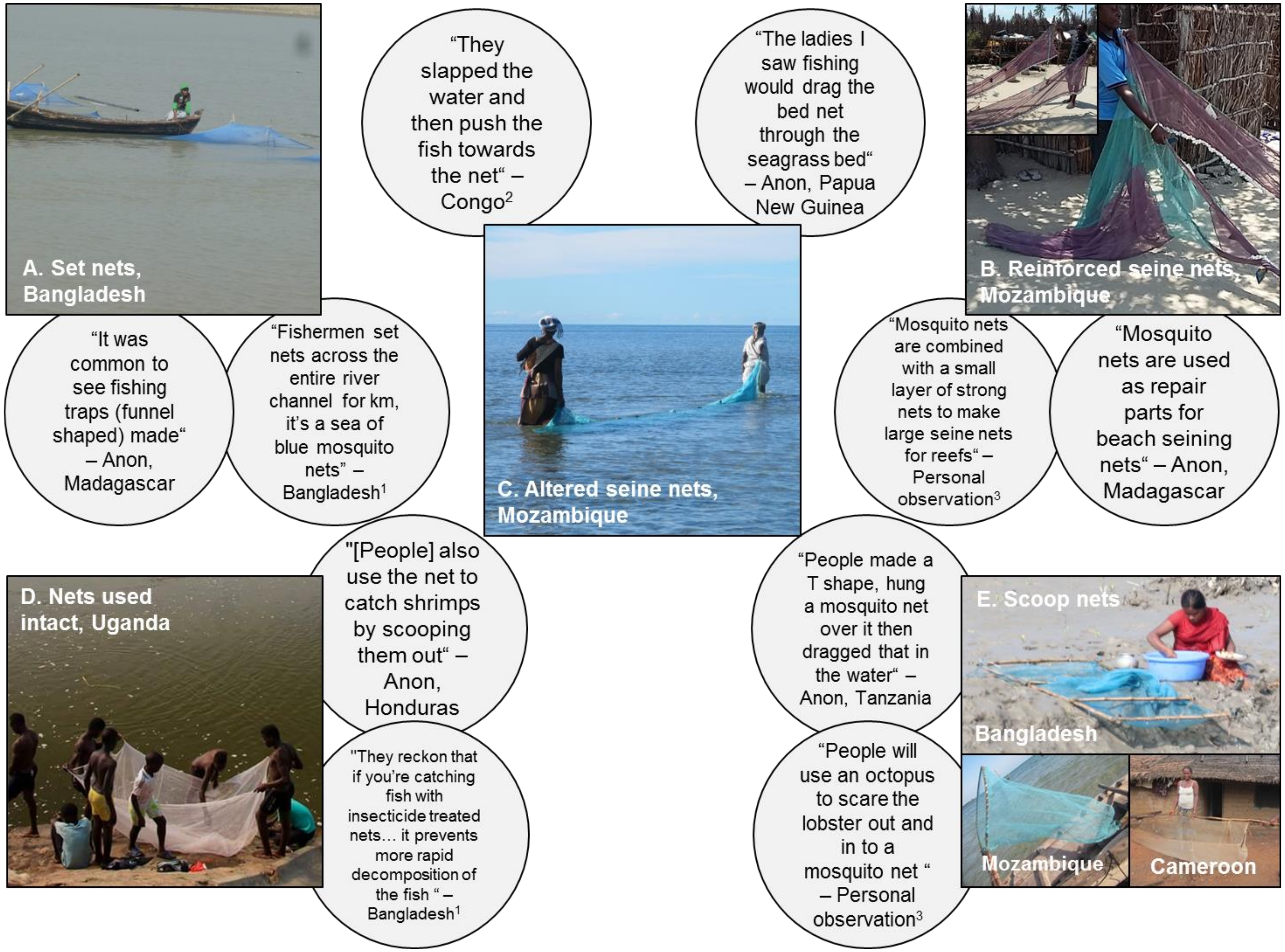

S7 Fig. Summary of additional information provided by respondents.

Photo credits: A) R. C. Browmick, B+C) R. Short, D) C. Hopkins, E) Bangladesh - N. Dewhurst-Richman, Mozambique – R. Short, Cameroon – J. Wright

[1]Nadia Dewhurst-Richman, Zoological Society of London; [2]Petra Lahann, German Development Corporation; [3]Rebecca Short, Imperial College London.
