# Supplementary material for: The use of mosquito nets in fisheries: A global perspective
Source: PLoS One. 2018 Jan 31;13(1):e0191519. doi: 10.1371/journal.pone.0191519 (PMC5791988; doi:10.1371/journal.pone.0191519)
Supplement: S1 Table — (PDF) [file pone.0191519.s008.pdf]

S1 Table: Predominant peer reviewed literature with direct reference to use of MNs for fishing at specific locations at time of questionnaire

Reference	Juvenile catch inferred	User group/s noted	Location	Marine/ freshwater	Potential impacts	Potentially detracting from bed coverage?
(Abbott and Campbell, 2009)	Yes	Women and children	Namibia (upper Zambezi floodplains)	Freshwater	Insecticide pollution	-
(Allan et al., 2012)	-	-	Chad	Freshwater	-	-
(Atkinson et al., 2009)	-	-	Solomon Islands	-		-
(Banek et al., 2010)	-	-	Liberia	-	-	Yes
(Bennett et al., 2012)	-	-	Sierra Leone	-	-	-
(Darkey and Turatsinze, 2014)	Yes	Artisanal fishermen	Mozambique, Beira	Marine	Declining catch; Damage to benthos	-
(Devi et al., 2013)	-	Men, women, children	India	Freshwater	-	-
(Endebu et al., 2015)	-	-	Ethiopia, Lake Zeway	Freshwater	-	-
(Halafo et al., 2004)	Yes	-	Mozambique, lake Niassa	Freshwater	Stock depletion	-
(Hamerlynck et al., 2011)	Yes	Women and children	Tanzania	Freshwater	-	-
(Jiddawi and Öhman, 2002)	Yes	Women	Tanzania	Marine	-	
(Kimirei et al., 2008)	Yes	-	Tanzania, Lake Tanganyika	Freshwater	Fishery collapse	-
(Koenker et al., 2013)	-	-	Tanzania, Zanzibar	Marine	-	-
(Larson et al., 2014)	-	-	Kenya, Lake Victoria	Freshwater	-	Yes
(Loll et al., 2013)	-	-	Senegal	-	-	-
(Lover et al., 2011)	-		Timor-Leste	-	-	Yes
(McLean et al., 2014)	Yes	-	Tanzania, Lake Tanganyika, DRC	Freshwater	Declining catch; Insecticide pollution; carcinogenic effects	Possibly - malaria rates unaffected
(Minakawa et al., 2008)	Not explicitly	-	Kenya, Lake Victoria	Freshwater	-	Yes
(Mosepele et al., 2009)	-	-	Botswana, Okavango delta	Freshwater	-	-
(Mushagalusa et al., 2014)	-	-	DRC, Lake Tanganyika	Freshwater	-	-
(Mutuku et al., 2013)	-	-	Kenya	Marine	-	Yes
(Okeyo et al., 2004)	-	Men and women	Namibia	Freshwater	-	-
(Quarcoopome et al., 2011)	Yes	-	Ghana	Freshwater	Stock depletion	-
(Siddique et al., 2013)	-	-	Bangladesh	Marine	-	-
(Sinha and Sinha, 2013)	-	-	India	Freshwater	Stock depletion	-
(Srivastava et al., 2002)	-	-	India	Freshwater	-	-
(Tynsong and Tiwari, 2008)	-	-	India	Freshwater	-	-
(van der Elst, 2003)	Yes	Women	Mozambique	Marine	Conflicts with commercial trawler fishery	-
(Tweddle et al., 2015)	-	-	Zambia, Malawi	Freshwater	-	-

Abbott, J.G., Campbell, L.M., 2009. Environmental histories and emerging fisheries management of the Upper Zambezi river floodplains. Conserv. Soc. 7, 83–99.

Allan, R., O'Reilly, L., Gilbos, V., Kilian, A., 2012. An Observational Study of Material Durability of Three World Health Organization–Recommended Long-Lasting Insecticidal Nets in Eastern Chad. Am. J. Trop. Med. Hyg. 87, 407–411. doi:10.4269/ajtmh.2012.11-0331

Atkinson, J.-A., Bobogare, A., Fitzgerald, L., Boaz, L., Appleyard, B., Toaliu, H., Vallely, A., 2009. A qualitative study on the acceptability and preference of three types of long-lasting insecticide-treated bed nets in Solomon Islands: implications for malaria elimination. Malar. J. 8, 119. doi:10.1186/1475-2875-8-119

Banek, K., Kilian, A., Allan, R., 2010. Evaluation of Interceptor long-lasting insecticidal nets in eight communities in Liberia. Malar J 9, 84.

Bennett, A., Smith, S.J., Yambasu, S., Jambai, A., Alemu, W., Kabano, A., Eisele, T.P., 2012. Household Possession and Use of Insecticide-Treated Mosquito Nets in Sierra Leone 6 Months after a National Mass-Distribution Campaign. PLoS ONE 7, e37927. doi:10.1371/journal.pone.0037927

Darkey, D., Turatsinze, R., 2014. Artisanal Fishing in Beira, Central Mozambique. J Hum Ecol 47, 317–328.

Devi, B.N., Mishra, S.K., Das, L., Pawar, N.A., Chanu, T.I., 2013. Traditional fishing methods in Central valley region of Manipur, India. Indian J. Tradit. Knowl. 12, 137–143.

Endebu, M., Lema, A., Genet, T., Mitike, A., 2015. Fisheries Baseline Survey Describing Status of Fisheries in Lake Zeway, Ethiopia. J. Fish. Livest. Prod. 03. doi:10.4172/2332-2608.1000129

Halafo, J.S., Hecky, R.E., Taylor, W.D., 2004. The artisanal fishery of Metangula, Lake Malawi/Niassa, East Africa. Afr. J. Aquat. Sci. 29, 83–90. doi:10.2989/16085910409503795

Hamerlynck, O., Duvail, S., Vandepitte, L., Kindinda, K., Nyingi, D.W., Paul, J.-L., Yanda, P.Z., Mwakalinga, A.B., Mgaya, Y.D., Snoeks, J., 2011. To connect or not to connect? Floods, fisheries and livelihoods in the Lower Rufiji floodplain lakes, Tanzania. Hydrol. Sci. J. 56, 1436–1451. doi:10.1080/02626667.2011.630002

Jiddawi, N.S., Öhman, M.C., 2002. Marine Fisheries in Tanzania. AMBIO J. Hum. Environ. 31, 518. doi:10.1639/0044-7447(2002)031[0518:MFIT]2.0.CO;2

Kimirei, I.A., Mgaya, Y.D., Chande, A.I., 2008. Changes in species composition and abundance of commercially important pelagic fish species in Kigoma area, Lake Tanganyika, Tanzania. Aquat. Ecosyst. Health Manag. 11, 29–35. doi:10.1080/14634980701881490

Koenker, H.M., Loll, D., Rweyemamu, D., Ali, A.S., 2013. A good night's sleep and the habit of net use: perceptions of risk and reasons for bed net use in Bukoba and Zanzibar. Malar J 12, 10–1186.

Larson, P.S., Minakawa, N., Dida, G.O., Njenga, S.M., Ionides, E.L., Wilson, M.L., 2014. Insecticide-treated net use before and after mass distribution in a fishing community along Lake Victoria, Kenya: successes and unavoidable pitfalls. Malar J 13, 466.

Loll, D.K., Berthe, S., Faye, S.L., Wone, I., Koenker, H., Arnold, B., Weber, R., 2013. User-determined end of net life in Senegal: a qualitative assessment of decision-making related to the retirement of expired nets. Malar J 12, 10–1186.

Lover, A.A., Sutton, B.A., Asy, A.J., Wilder-Smith, A., 2011. An exploratory study of treated-bed nets in Timor-Leste: patterns of intended and alternative usage. Malar. J. 10, 199.

McLean, K.A., Byanaku, A., Kubikonse, A., Tshowe, V., Katensi, S., Lehman, A.G., 2014. Fishing with bed nets on Lake Tanganyika: a randomized survey. Malar J 13, 10–1186.

Minakawa, N., Dida, G.O., Sonye, G.O., Futami, K., Kaneko, S., 2008. Unforeseen misuses of bed nets in fishing villages along Lake Victoria. Malar. J. 7, 165. doi:10.1186/1475-2875-7-165

Mosepele, K., Moyle, P.B., Merron, G.S., Purkey, D.R., Mosepele, B., 2009. Fish, Floods, and Ecosystem Engineers: Aquatic Conservation in the Okavango Delta, Botswana. BioScience 59, 53–64. doi:10.1525/bio.2009.59.1.9

Mushagalusa, C.D., Nshombo, M., Lushombo, M., 2014. Littoral fisheries on Cichlidae (Pisces) from the northwestern part of Lake Tanganyika, East Africa. Aquat. Ecosyst. Health Manag. 17, 41–51.

Mutuku, F.M., Khambira, M., Bisanzio, D., Mungai, P., Mwanzo, I., Muchiri, E.M., King, C.H., Kitron, U., 2013. Physical condition and maintenance of mosquito bed nets in Kwale County, coastal Kenya. Malar. J. 12, 46. doi:10.1186/1475-2875-12-46

Okeyo, D.O., Mubita, G., Harris, T.K., Sahombu, D.E., Namundjanga, J., Mulonga, S., Kapirika, S., 2004. Indigenous names of fish and fishing gear in the Cuvelai, Kavango and Caprivi regions of Namibia. Afr. J. Aquat. Sci. 29, 249–258. doi:10.2989/16085910409503817

Quarcoopome, T., Amevenku, F., Ofori-Danson, P., 2011. Changes in the fish community of the Kpong Headpond, lower Volta River, Ghana after 25 years of impoundment. Rev. Biol. Trop. 59, 1685–1696.
Siddique, A.B., Saha, D., Rahman, M., Hossain, M.B., 2013. Fishing gears of the Meghna river estuary of Chandpur region, Bangladesh.
Sinha, Rajesh K., Sinha, R. K., 2013. Diversity of selective and non-selective fishing gears and their impact on Ganga fishery in Bihar. Int. J. Bioassays 2, 739–750.
Srivastava, S.K., Sarkar, U.K., Patiyal, R.S., 2002. Fishing methods in streams of the Kumaon Himalayan region of India. Asian Fish. Sci. 15, 347–356.
Tweddle, D., Cowx, I.G., Peel, R.A., Weyl, O.L.F., 2015. Challenges in fisheries management in the Zambezi, one of the great rivers of Africa. Fish. Manag. Ecol. 22, 99–111. doi:10.1111/fme.12107
Tynsong, H., Tiwari, B.K., 2008. Traditional knowledge associated with fish harvesting practices of War Khasi of Meghalaya. Indian J. Tradit. Knowl. 7, 618–623.
van der Elst, 2003. Local solutions to challenges of West Indian Ocean fi sheries development. NAGA WorldFish Cent. Q. 26, 14–17.
