# Supplementary material for: The use of mosquito nets in fisheries: A global perspective
Source: PLoS One. 2018 Jan 31;13(1):e0191519. doi: 10.1371/journal.pone.0191519 (PMC5791988; doi:10.1371/journal.pone.0191519)
Supplement: S2 Table — (PDF) [file pone.0191519.s009.pdf]

S2 Table. Families identified by observations in MNF catch. NB – maturity status was not individually obtained for families so groupings are based on general associations at adult life stages.

Reef/seagrass associated	Pelagic/neritic associated	Freshwater
Acanthuridae	Ariidae	Alestidae
Balistidae	Atherinidae	Aplocheilidae
Caesonidae	Carangidae	Characidae
Chaetodonidae	Chanidae	Cichlidae
Epinephelinae	Clupidae	Clupidae
Gobiidae	Drepaneidae	Cyprinidae
Haemulidae	Elopidae	Gobiidae
Holocentridae	Engraulidae	
Labridae	Gerridae	
Lethrinidae	Leiognathidae	
Lutjanidae	Mugilidae	
Mullidae	Scombridae	
Platycephalidae	Sparidae	
Pomacentridae		
Scaridae		
Sciaenidae		
Serranidae		
Siganidae		
Syngnathidae		
Tetradaontidae		
