# Supplementary material for: The use of mosquito nets in fisheries: A global perspective
Source: PLoS One. 2018 Jan 31;13(1):e0191519. doi: 10.1371/journal.pone.0191519 (PMC5791988; doi:10.1371/journal.pone.0191519)
Supplement: S1 Text — (PDF) [file pone.0191519.s010.pdf]

S1 Text. Mosquito net fishing questionnaire as presented on www.qualtrics.com

Introduction to online questionnaire

Thank you for participating in this survey about the gears used in small-scale artisanal fishing in developing countries. We are particularly interested in the degree to which bed nets distributed for malaria control are being used in fishing in different parts of the world. There are two groups of people who we are targeting this survey towards, anyone who has knowledge of small-scale and artisanal fishing, and anyone who is involved in malaria control efforts in fishing areas of developing countries. We would be very grateful if you could complete the survey if you belong to one of these two groups regardless of whether you have observed bed nets being used in fishing because we are interested in both positive and negative observations of net use. The survey should only take about 15 minutes to complete and it should take less time if you haven't seen fishing with bed nets.

There have been a number of reports of bed nets being used for fishing, but these have been anecdotal or local. This study will give the first global perspective on the use of insecticide-treated bed nets and long-lasting insecticide-treated bed nets for fishing. This will increase understanding of the scale and methods of fishing with bed nets and its role in people’s livelihoods so that we can provide advice on how to manage this growing phenomenon.

This questionnaire is part of an MSc research project by Rajina Gurung, from Imperial College London, in partnership with Zoological Society of London. The information you give will provide a valuable insight into this extremely poorly understood and documented issue. Your responses are anonymous and confidential, and general trends will be reported rather than specifics. We will be careful to ensure that our research does not harm local people in the areas of study, and that we maintain the highest ethical standards.

Are you happy to proceed on this basis?

- ☐ Yes
- ☐ No

Please specify which area you are most familiar with to take you to the relevant survey

- ☐ Small-scale and artisanal fishing
- ☐ Malaria control efforts

Survey targeted at individuals with knowledge of small-scale artisanal fishing

1. Country in which you are based:
2. Which country are you originally from?
3. Organisation:
4. Position:
5. Would you say your work is predominantly...

- ☐ Development focused
- ☐ Conservation focused
- ☐ Fisheries focused
- ☐ Other (please describe)

Please think about specific locations where you have had the opportunity to observe fishing practices for a substantial amount of time, either currently or in the past. The scale of location we are ideally looking for is at a village level but it could also be an area of coastline, river, lake, fishing location or a region. I will ask you to complete a separate survey for each location for which you feel able to give information

6. Please give the name of the location:

7. Country:
8. Type of location:
   - ☐ Coastal
   - ☐ Lake
   - ☐ River
   - ☐ Wetland
   - ☐ Other (please specify)
9. Location information
   - ☐ Size of location (please specify in km2 if known):
   - ☐ Lat/Long (if known):
   - ☐ Human population density (if known):
10. What kind of work do you do that takes you to this location?
    - ☐ Mainly field based
    - ☐ Mainly office based
    - ☐ A mix of field and office based
    - ☐ Other (please describe)
11. In which year did you start observing fishing at this location?
12. Are you still observing fishing at this location?
13. If no, for how many years did you observe fishing at this location?
    - ☐ 0-1 years
    - ☐ 2-5 years
    - ☐ 5-10 years
    - ☐ 10 years +
14. How well would you say you know the area and its fishing practices?
    - ☐ Very well
    - ☐ Moderately well
    - ☐ Not very well
    - ☐ Don't know
15. What are the predominant fishing activities in the area? Please tick all that apply
    - ☐ Commercial fishing
    - ☐ Artisanal fishing (for income)
    - ☐ Subsistence fishing (for domestic consumption)
    - ☐ Don't know

Picture A shows an untransformed bed net and picture B shows a bed net used for fishing. Nets made out of bed-netting have very small mesh size less than 3mm. Nets may be of variable shape, size and colour.

Picture A

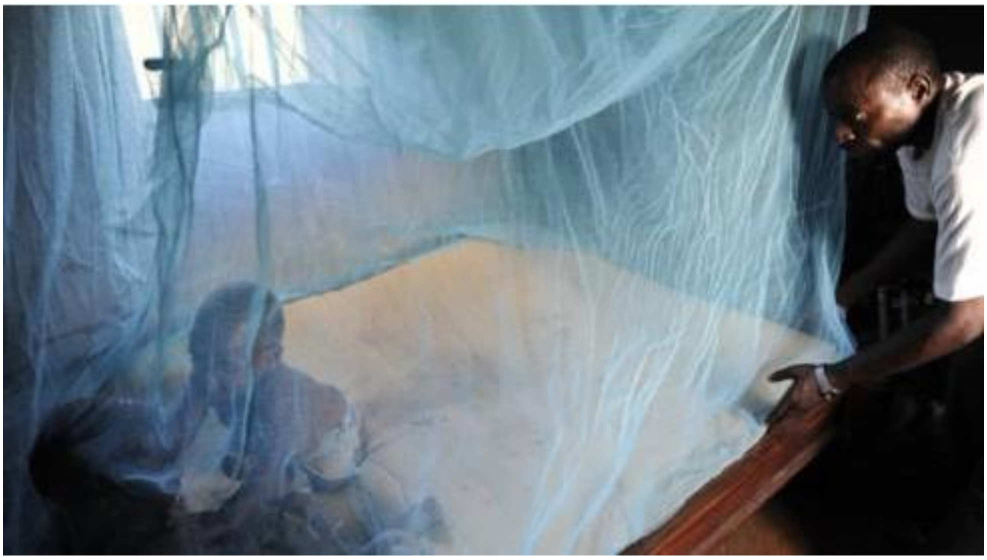

Picture B

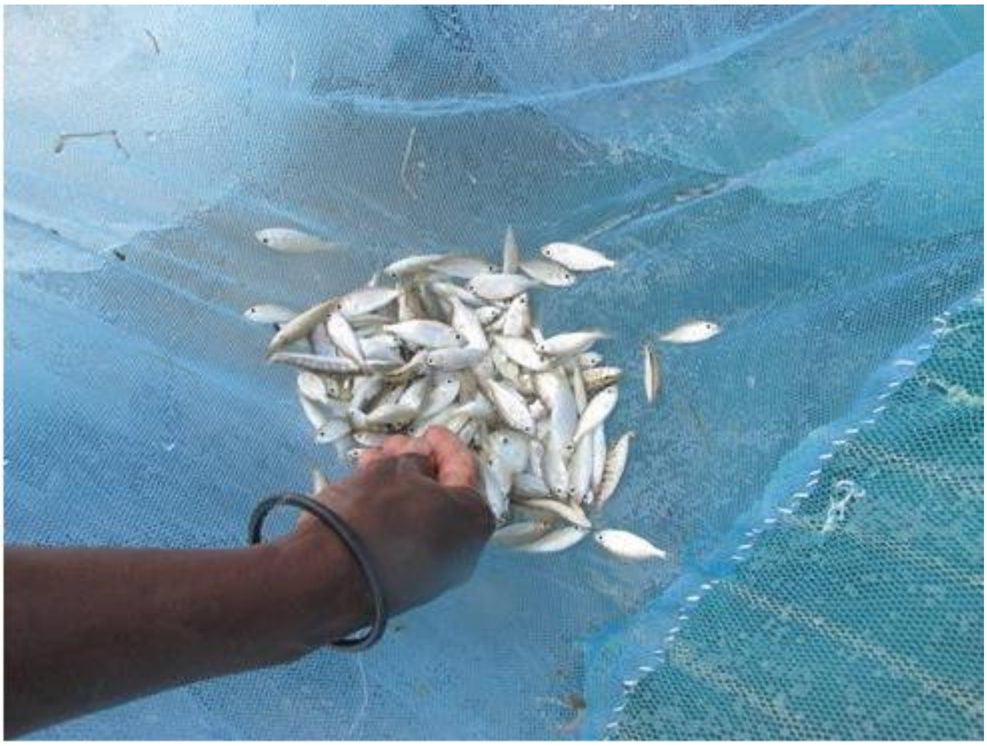

16. Have you ever observed fishing with bed nets at this location?
    - ☐ Yes (if you have photographic evidence please send images to email address at end of survey)
    - ☐ No

17. How frequently have you observed this happening, on average?
    - ☐ Every day
    - ☐ More than once a week
    - ☐ Once a week
    - ☐ Once a month
    - ☐ Occasionally
    - ☐ Other (please describe)

☐ Don’t know

18. What is the temporal pattern of bed net fishing?
    ☐ Year-round
    ☐ Seasonal (please describe)
    ☐ Tide-dependent (please describe)
    ☐ Don’t know
19. In which of the following habitats have you seen fishing with bed nets? (Please select all that apply)
    ☐ From the beach
    ☐ At sea
    ☐ Lake
    ☐ River
    ☐ Local stream
    ☐ Coral reef
    ☐ Seagrass beds
    ☐ Mangroves
    ☐ Other (please describe)
    ☐ Don’t know
20. How have you observed fishers fishing with bed nets? (Please select all that apply)
    ☐ On foot
    ☐ On a dugout canoe
    ☐ On a sail boat
    ☐ On a motorised vessel
    ☐ On a commercial fishing vessel
    ☐ Other (please describe)
    ☐ Don’t know
21. How are fishers using bed nets to catch fish? (Please select all that apply)
    ☐ Use the bed net on its own to scoop fish out
    ☐ Sew the bed nets together to make a large fishing net
    ☐ Sew the bed net into the cod end of a larger fishing net
    ☐ Use the insecticide in the bed net to catch fish
    ☐ Other (please describe)
    ☐ Don’t know
22. Who have you observed fishing with bed nets?

	Never	Sometimes	Often
Children	☐	☐	☐
Working-age women	☐	☐	☐
Working-age men	☐	☐	☐
Elderly	☐	☐	☐

23. Are bed nets predominately used by:
    ☐ Experienced fishers
    ☐ Part time fishers
    ☐ Inexperienced fishers
    ☐ Don’t know

    Why do you think this?

24. What other occupations do bed net fishers have?
    ☐ Fishing with other gear
    ☐ Agriculture
    ☐ Small business owner
    ☐ Casual labour

- ☐ Other, please describe
- ☐ Don’t know

25. In your opinion do you think people fish with bed nets…
    - ☐ For domestic consumption
    - ☐ To sell the fish
    - ☐ A mix of both
    - ☐ Other, please describe
    - ☐ Don’t know
26. Which species are predominately caught in bed nets? (tick the main ones)
    - ☐ Reef fish (please specify main species if known)
    - ☐ Pelagic fish (please specify main species if known)
    - ☐ Octopus
    - ☐ Molluscs (please specify)
    - ☐ Crustaceans (please specify)
    - ☐ Other (please describe)
    - ☐ Don’t know
27. Have you observed juvenile fish being caught in bed nets?
    - ☐ Yes (please describe how you know)
    - ☐ No
    - ☐ Don’t know
28. Have you observed threatened or high value species being caught with bed nets?
    - ☐ Yes (please describe which species if known)
    - ☐ No
    - ☐ Don’t know
29. Are you aware of any legal restrictions to the use of bed nets for fishing in this location?
    - ☐ Yes (please describe, e.g. if legal or illegal throughout the year or at certain times of the year)
    - ☐ No

    If YES, are you aware of any active enforcement in the area and by whom?

30. Are you aware of any local customs or rules which relate to the use of bed nets for fishing?
    - ☐ Yes, please describe
    - ☐ No

    If YES, are you aware of any active enforcement in the area and by whom?

31. How do bed nets relate to other gears?
    - ☐ Used instead of existing gears
    - ☐ Used additionally to existing gears
    - ☐ Other (please describe)
    - ☐ Don't know
32. When did you first notice the use of bed nets for fishing?
    - ☐ They were already being used when I arrived
    - ☐ They came into use during my time at the location
    - ☐ Not sure
33. In what year did you first notice bed nets being used for fishing?
34. Do you know if there been any major change in prevalence of bed net use for fishing over time?
    - ☐ Yes (please describe)
    - ☐ No
35. Do you know what proportion of the population currently fish with bed nets?
    - ☐ Yes (please specify)
    - ☐ No
36. Why might a household choose to use bed nets for fishing (give up to 3 reasons)?
37. Why might a household not choose to use bed nets for fishing (give up to 3 reasons)?

Thank you so much for your input so far. Just a few short questions left on the distribution of bed nets in the location.

38. Have bed nets been distributed for malaria prevention in the location?
    - ☐ Yes
    - ☐ No
39. When was the last distribution of bed nets in the location?
    - ☐ Within the last year
    - ☐ 2-3 years ago
    - ☐ 4-5 years ago
    - ☐ 6-7 years ago
    - ☐ 8-9 years ago
    - ☐ 10 years +
    - ☐ Don’t know
40. Do you know who the last organisation was who distributed the nets?
    - ☐ Yes (please specify)
    - ☐ No
    - ☐ Don't know
41. How are nets reaching the area? Please rank in order of prevalence
    - ☐ Distributed at health clinics (please specify if distributed for free or at a subsidised price if known)
    - ☐ Distributed at maternity clinics (please specify if distributed for free or at a subsidised price if known)
    - ☐ Distributed from government campaigns (please specify if distributed for free or at a subsidised price if known)
    - ☐ Sold at local shops
    - ☐ Sold from traders
    - ☐ Received from relatives / family
    - ☐ Distributed for free at schools
    - ☐ Other (please describe)
    - ☐ Don’t know
42. Do you know what happens to bed nets after they are no longer usable for malaria control, in order of prevalence? (please select all that apply)
    - ☐ Thrown away
    - ☐ Destroyed by burning
    - ☐ Used for fishing
    - ☐ Alternative uses e.g. crop cover, wedding dresses etc (please describe)
    - ☐ Other (please describe)
    - ☐ Don’t know

Contact email address or telephone number (optional if you have witnessed fishing with bed nets or have an opinion on the matter and would like to discuss further

Thank you very much for your time. Please use the space below to add any other comments. If you would like a copy of my results please specify in the space below and put in your email address if you haven't included it already. If you have any photographic evidence of fishing with bed nets, other information to share (e.g. relevant reports) or would like to discuss this further, please email me at: rajina.gurung14@imperial.ac.uk.

If you feel you are able to give more information about fishing practices in another location please select the ‘yes, I have more information to give on another location’ box to fill out another survey for a separate location. If you would like to end the survey please select ‘no, I do not have any more information to give on another location.’

- ☐ Further comments
- ☐ Yes, I have more information to give on another location

☐ No, I do not have any more information to give on another location

Survey targeted at individuals involved in malaria control efforts

1. Country in which you are based:
2. Which country are you originally from?
3. Organisation:
4. Position:
5. Would you say your work is predominantly...
   ☐ Development focused
   ☐ Health focused
   ☐ Other (please describe)

Please think about specific distinct locations where you have had the opportunity to observe bed net distributions or been involved in malaria control efforts in fishing areas of developing countries, either currently or in the past. A location could be a village, area of coastline / lake / river, fishing location or a region. I will ask you to complete a separate survey for each location for which you feel able to give information.

6. Please give the name of the location:
7. Country:
8. Type of location:
   ☐ Coastal
   ☐ Lake
   ☐ River
   ☐ Wetland
   ☐ Other (please specify)
9. Location information
   ☐ Size of location (please specify in km2 if known):
   ☐ Lat/Long (if known):
   ☐ Human population density (if known):
10. What kind of work do you do that takes you to this location?
    ☐ Mainly field based
    ☐ Mainly office based
    ☐ A mix of field and office based
    ☐ Other (please describe)
11. How long have you worked in this location?
    ☐ 0-1years
    ☐ 2-5 years
    ☐ 5-10 years
    ☐ 10 years +
12. How well would you say you know the area?
    ☐ Very well
    ☐ Moderately well
    ☐ Not very well
    ☐ Don’t know
13. Have bed nets been distributed for malaria prevention in the location?
    ☐ Yes
    ☐ No
14. When was the last distribution of bed nets in the location?
    ☐ Within the last year
    ☐ 2-3 years ago
    ☐ 4-5 years ago
    ☐ 6-7 years ago
    ☐ 8-9 years ago

- ☐ 10 years +
- ☐ Don't know

15. Do you know which brand of insecticide-treated bed net or long lasting insecticide treated bed net has been distributed or is prevalent in the area? (e.g. Olyset, PermaNet, Netprotect, Duranet, Inceptor etc)
    - ☐ Yes (please specify)
    - ☐ No
16. Do you have any information on the uptake (number of bed nets distributed) of bed nets from the last distribution campaign?
    - ☐ Yes (please describe)
    - ☐ No
17. Do you have any information on the proportion of individuals sleeping under bed nets in this location?
    - ☐ Yes (please describe)
    - ☐ No
18. If relevant, do you have any specific targets for uptake (number of bed nets distributed) and coverage (number of bed nets being used on beds) of bed nets?
    - ☐ Yes (please describe)
    - ☐ No
    - ☐ Don't know
19. If relevant, what is your organisations policy on disposal of old bed nets?
    - ☐ Replacing old net for new net
    - ☐ No disposal
    - ☐ Self-disposal by recipient
    - ☐ Other (please specify)
    - ☐ Don't know
20. How are nets reaching the area? Please rank in order of prevalence
    - ☐ Distributed at health clinics (please specify if distributed for free or at a subsidised price if known)
    - ☐ Distributed at maternity clinics (please specify if distributed for free or at a subsidised price if known)
    - ☐ Distributed from government campaigns (please specify if distributed for free or at a subsidised price if known)
    - ☐ Sold at local shops
    - ☐ Sold from traders
    - ☐ Received from relatives / family
    - ☐ Distributed for free at schools
    - ☐ Other (please describe)
    - ☐ Don't know
21. Do you know what happens to bed nets after they are no longer usable for malaria control, in order of prevalence? (please select all that apply)
    - ☐ Thrown away
    - ☐ Destroyed by burning
    - ☐ Used for fishing
    - ☐ Alternative uses e.g. crop cover, wedding dresses etc (please describe)
    - ☐ Other (please describe)
    - ☐ Don't know
22. Where is this information coming from? E.g. own research, malaria indicator survey etc (please specify)

Picture A shows an untransformed bed net and picture B shows a bed net used for fishing. Nets made out of bed-netting have very small mesh size less than 3mm. Nets may be of variable shape, size and colour.

Picture A

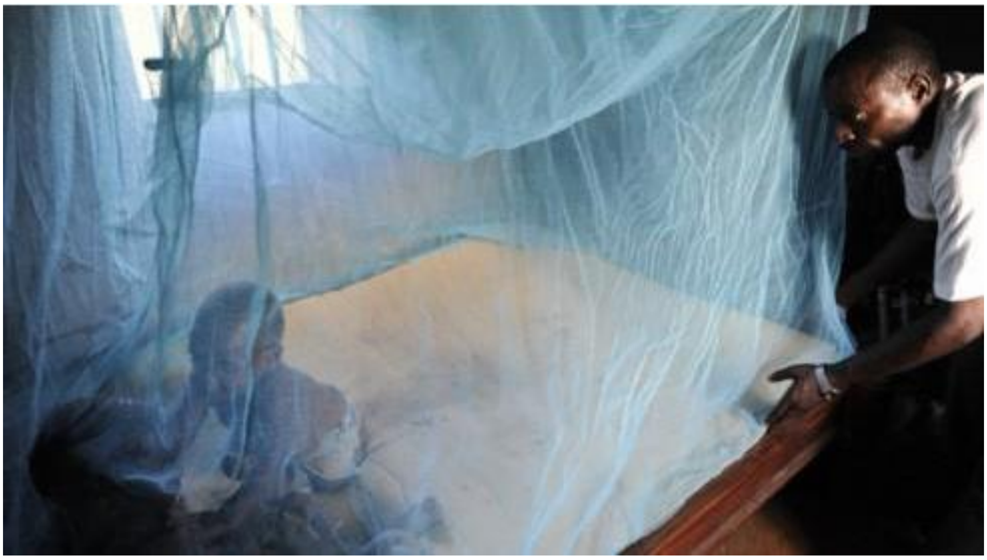

Picture B

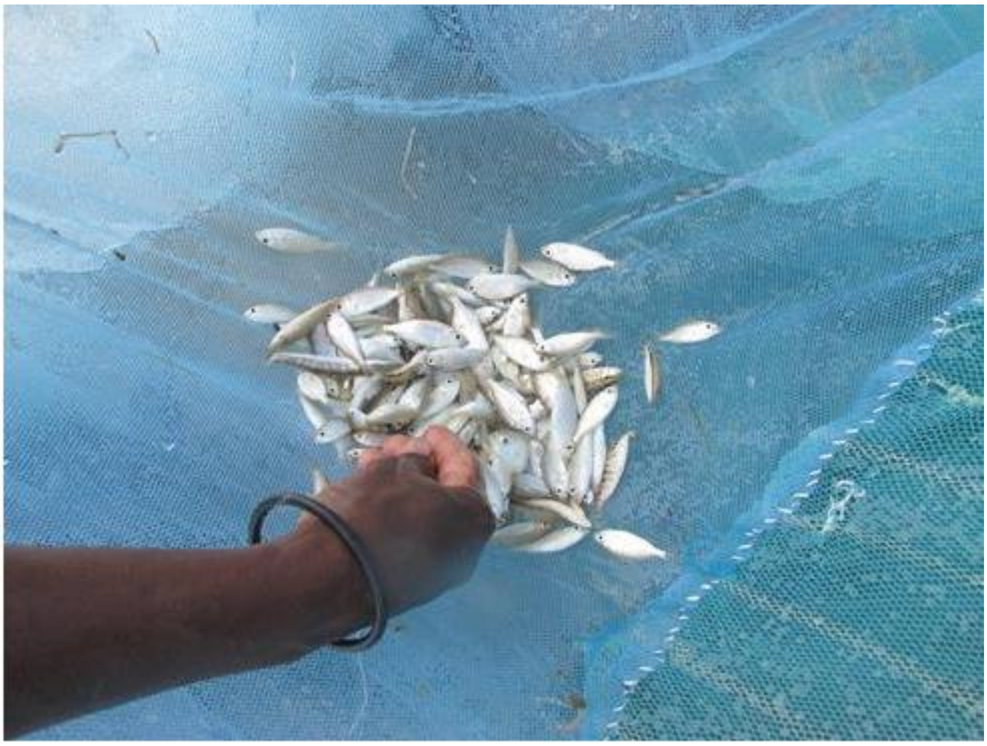

23. Have you ever observed fishing with bed nets at this location?
    - □ Yes (if you have photographic evidence please send images to email address at end of survey)
    - □ No

24. How frequently have you observed this happening, on average?
    - ☐ Every day
    - ☐ More than once a week
    - ☐ Once a week
    - ☐ Once a month
    - ☐ Occasionally
    - ☐ Other (please describe)
    - ☐ Don't know
25. What is the temporal pattern of bed net fishing?
    - ☐ Year-round
    - ☐ Seasonal, please describe
    - ☐ Tide-dependent (please describe)
    - ☐ Don't know
26. In which of the following habitats have you seen fishing with bed nets? (Please select all that apply)
    - ☐ From the beach
    - ☐ At sea
    - ☐ Lake
    - ☐ River
    - ☐ Local stream
    - ☐ Coral reef
    - ☐ Seagrass beds
    - ☐ Mangroves
    - ☐ Other (please describe)
    - ☐ Don't know
27. How have you observed fishers fishing with bed nets? (Please select all that apply)
    - ☐ On foot
    - ☐ On a dugout canoe
    - ☐ On a sail boat
    - ☐ On a motorised vessel
    - ☐ On a commercial fishing vessel
    - ☐ Other (please describe)
    - ☐ Don't know
28. Who have you observed fishing with bed nets?

	Never	Sometimes	Often
Children	☐	☐	☐
Working-age women	☐	☐	☐
Working-age men	☐	☐	☐
Elderly	☐	☐	☐

29. How are fishers using bed nets to catch fish? (Please select all that apply)
    - ☐ Use the bed net on its own to scoop fish out
    - ☐ Sew the bed nets together to make a large fishing net
    - ☐ Sew the bed net into the cod end of a larger fishing net
    - ☐ Use the insecticide in the bed net to catch fish
    - ☐ Other (please describe)
    - ☐ Don't know
30. Are bed nets predominately used by:
    - ☐ Experienced fishers
    - ☐ Part time fishers
    - ☐ Inexperienced fishers
    - ☐ Don't know

    Why do you think this?

31. What other occupations do bed net fishers have?
    - ☐ Fishing with other gear
    - ☐ Agriculture
    - ☐ Small business owner
    - ☐ Casual labour
    - ☐ Other, please describe
    - ☐ Don’t know
32. In your opinion do you think people fish with bed nets…
    - ☐ For domestic consumption
    - ☐ To sell the fish
    - ☐ A mix of both
    - ☐ Other, please describe
    - ☐ Don’t know
33. When did you first notice the use of bed nets for fishing?
    - ☐ They were already being used when I arrived
    - ☐ They came into use during my time at the location
    - ☐ Not sure
34. In what year did you first notice bed nets being used for fishing?
35. Do you know if there been any major change in prevalence of bed net use for fishing over time?
    - ☐ Yes (please describe)
    - ☐ No
36. Do you know what proportion of the population currently fish with bed nets?
    - ☐ Yes (please specify)
    - ☐ No
37. Do you think the alternative use of bed nets is having an impact on malaria?
    - ☐ Yes
    - ☐ No
    - ☐ Don’t know

    Why do you think this?

38. If relevant, when distributing bed nets does your organisation have any procedures specifically to prevent the non-use or alternative use of bed nets?
    - ☐ Yes (please describe)
    - ☐ No
    - ☐ Don’t know

Contact email address or telephone number (optional if you have witnessed fishing with bed nets or have an opinion on the matter and would like to discuss further

Thank you very much for your time. Please use the space below to add any other comments. If you have any photographic evidence of fishing with bed nets, other information to share (e.g. relevant reports) or would like to discuss this further, please email me at: rajina.gurung14@imperial.ac.uk.

If you feel you are able to give more information on bed net distributions or malaria control efforts in another fishing area in a developing country please select the 'yes, I have more information to give' box to fill out another survey for the separate location. If you would like to end the survey please select 'no, I do not have any more information to give on another location.'

- ☐ Further comments
- ☐ Yes, I have more information to give on another location
- ☐ No, I do not have any more information to give on another location
