# Supplementary material for: The use of mosquito nets in fisheries: A global perspective
Source: PLoS One. 2018 Jan 31;13(1):e0191519. doi: 10.1371/journal.pone.0191519 (PMC5791988; doi:10.1371/journal.pone.0191519)
Supplement: S2 Fig — (PDF) [file pone.0191519.s002.pdf]

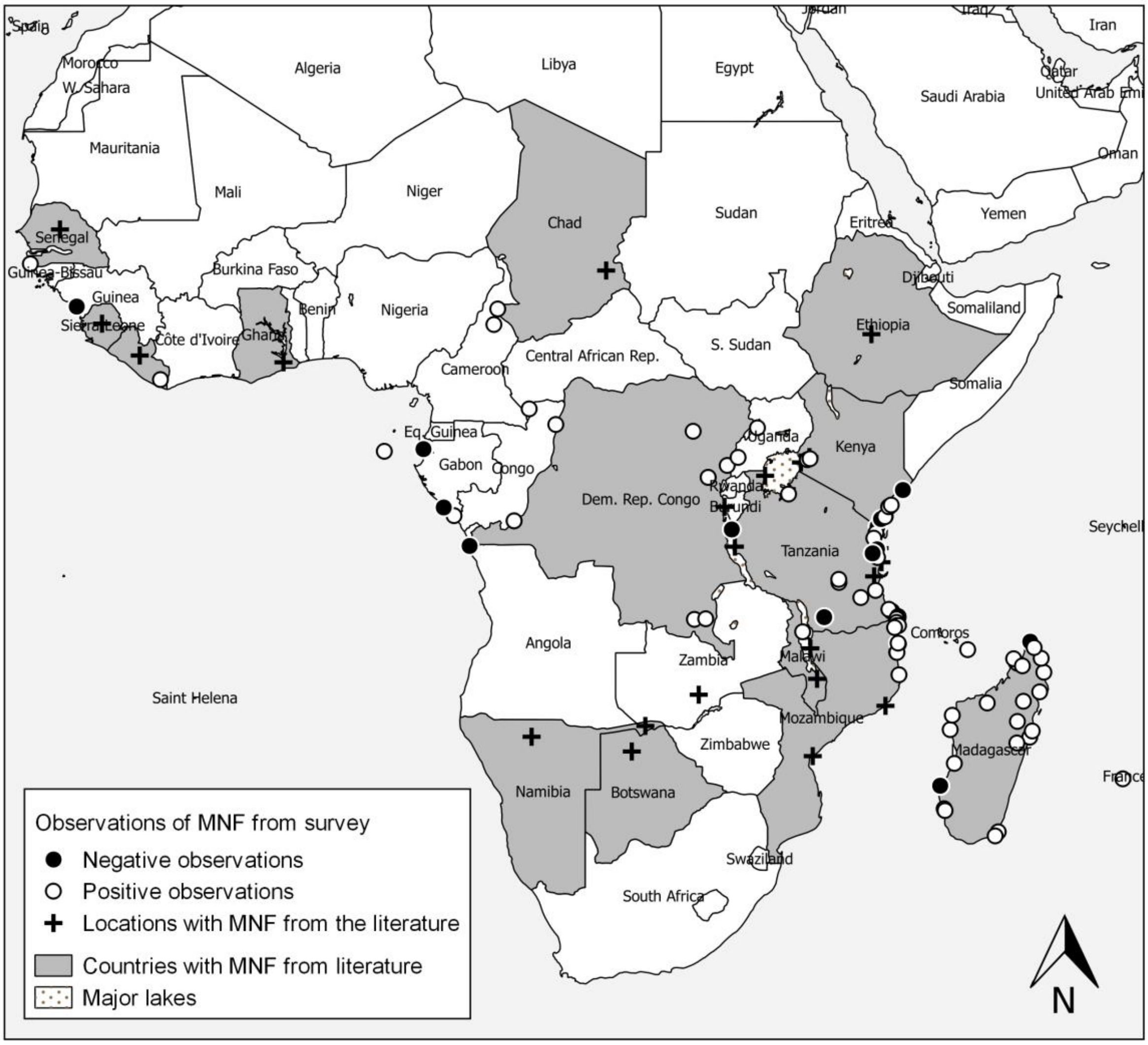

S2 Fig. Map of survey responses in Africa showing positive and negative reports of MNF
