# Supplementary material for: The use of mosquito nets in fisheries: A global perspective
Source: PLoS One. 2018 Jan 31;13(1):e0191519. doi: 10.1371/journal.pone.0191519 (PMC5791988; doi:10.1371/journal.pone.0191519)
Supplement: S2 Text — (PDF) [file pone.0191519.s011.pdf]

S2 Text - Additional MNF demographics and legislation results.

Observed restrictions on MNF

Fifty (38%) of the observations confirmed the existence of some kind of formal legal local or national restriction with a bearing on MNF activity, while two stated that restrictions did not exist. Of the restrictions, two (in 2 countries) were seasonal, the remaining 48 (12 countries) were year-round. Of the 19 respondents who were able to elaborate, 11 reported that they were based on existing laws concerning mesh size limits, six were specific laws for mosquito nets and two were based on seasonal management. A further 11 respondents reported some kind of local and/or informal restrictions such as Malagasy 'Dinas' (community-enforced rules), however few respondents were able to provide further detail.

Demographics of MN users

The reported frequency of engagement in MNF varied significantly across demographic groups, with women most commonly reported as engaging in MNF 'often', and men, children and the elderly most commonly reported as engaging in MNF 'sometimes' (S6 Fig, X-squared = 38.94, df = 6, Cramer's V = 0.27, p = <0.001). There was no significant regional difference in this trend (omitting Oceania and the Americas due to low response rates; X-squared = 4.67, df = 6, Cramer's V = 0.10, p-value = 0.59).

Thirty five percent of observations reported those engaging in MNF to be experienced fishers, 43% part time and 21% inexperienced fishers. In terms of occupational diversity, of 128 observations 28% reported that MN fishers also fish with other gears, 34% agriculture, 15% small business ownership and 23% casual labour. Other observations included formal employment and tourism.
